# A Review on Metal- and Metal Oxide-Based Nanozymes: Properties, Mechanisms, and Applications

**DOI:** 10.1007/s40820-021-00674-8

**Published:** 2021-07-09

**Authors:** Qianwen Liu, Amin Zhang, Ruhao Wang, Qian Zhang, Daxiang Cui

**Affiliations:** 1 Institute of Nano Biomedicine and Engineering, Shanghai Engineering Research Center for Intelligent Diagnosis and Treatment Instrument, Department of Instrument Science and Engineering, School of Electronic Information and Electrical Engineering, Shanghai Jiao Tong University, 800 Dongchuan RD, Shanghai, 200240 People’s Republic of China; 2 Institute of Nano Biomedicine, National Engineering Research Center for Nanotechnology, 28 Jiangchuan Easternroad, Shanghai, 200241 People’s Republic of China

**Keywords:** Metal- and metal oxide-based nanozymes, Intrinsic properties, Catalytic mechanism, Applications

## Abstract

Since the ferromagnetic (Fe_3_O_4_) nanoparticles were firstly reported to exert enzyme-like activity in 2007, extensive research progress in nanozymes has been made with deep investigation of diverse nanozymes and rapid development of related nanotechnologies. As promising alternatives for natural enzymes, nanozymes have broadened the way toward clinical medicine, food safety, environmental monitoring, and chemical production. The past decade has witnessed the rapid development of metal- and metal oxide-based nanozymes owing to their remarkable physicochemical properties in parallel with low cost, high stability, and easy storage. It is widely known that the deep study of catalytic activities and mechanism sheds significant influence on the applications of nanozymes. This review digs into the characteristics and intrinsic properties of metal- and metal oxide-based nanozymes, especially emphasizing their catalytic mechanism and recent applications in biological analysis, relieving inflammation, antibacterial, and cancer therapy. We also conclude the present challenges and provide insights into the future research of nanozymes constituted of metal and metal oxide nanomaterials.

## Highlights

- The characteristics of metal- and metal oxide-based nanozymes with diverse construction are dissertated.
- The intrinsic properties and catalytic mechanism of metal- and metal oxide-based nanozymes are discussed.
- The recent applications of metal- and metal oxide-based nanozymes in biological analysis, relieving inflammation, antibacterial, and cancer therapy are reviewed.

## Graphical abstract

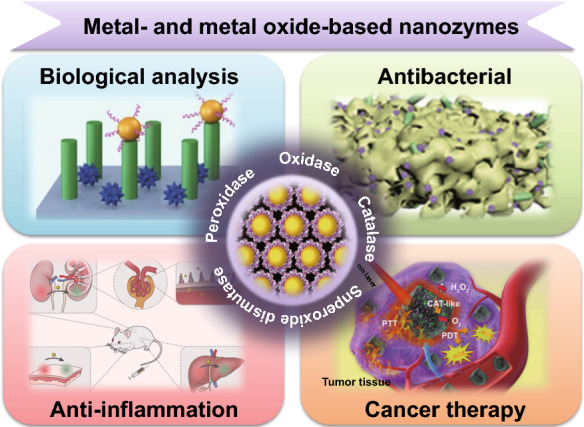

## Introduction

Enzymes are environmentally friendly biomaterials with remarkable catalytic efficiency and substrate specificity produced by living cells [1, 2]. Most of the natural enzymes are proteins, while a small part are RNA. The past decades have witnessed the extensive progress of biological enzymes in biology, medicine, chemistry, and industrial science [3]. Nevertheless, the complicated preparation procedure, unstable catalytical activity and intrinsic environmental sensitivity have restricted the scalable utilization of natural enzymes [4, 5]. Therefore, the exploration of alternative artificial enzymes to overcome shortcomings of natural catalysts has become an issue of increasing concern.

**Fig. 1:**
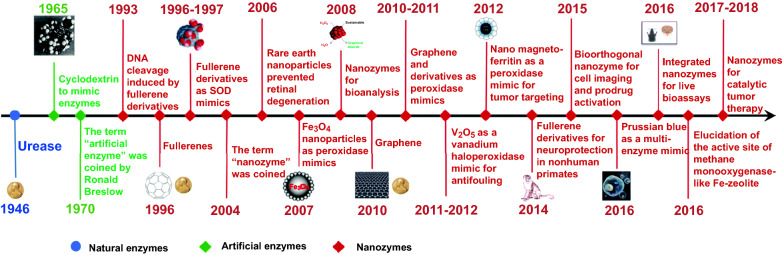
A brief timeline for the evolution of artificial enzymes and natural enzymes. Reproduced from Ref. [22] with permission

The evolution of nanotechnology and biology provides a bridge toward novel artificial enzymes. After the pioneering work of Gao et al. [6] reporting ferromagnetic (Fe_3_O_4_) nanoparticles (NPs) with enzyme-mimicking property in 2007, a bunch of nanozymes have been demonstrated as natural catalysts mimics. For instance, Au@Co–Fe hybrid NPs [7], CuCo_2_S_4_ NPs [8], MnO_2_ nanowires (NWs) [9], Pt nanoclusters (NCs) [10], Au@Pt nanorods (NRs) [11], and carboxyl-modified graphene oxide (GO–COOH) [12] have been reported as peroxidase (POD) mimics. Nanozymes with multi-enzyme-type activities (e.g., Co(OH)_2_/FeOOH/WO_3_ ternary nanoflowers [13], AuNPs [14, 15], Co_3_O_4_ NPs [16], AgPt NPs [17], N-doped sponge-like carbon spheres [18], Mn_3_O_4_ NPs [19]) have been exploited in diverse investigation. Up to date, more than 540 types of nanozymes have been synthesized by over 350 research laboratories from 30 countries [20]. Generally, existing nanozymes are affiliated with two categories, namely oxidoreductase family and hydrolase family. Carbon-based materials, metal, and transition metal compounds are the most common nanozyme composition materials [21]. Wu et al. reviewed the history of nanozyme and draw a brief timeline for the evolution of artificial enzymes and natural enzymes (Fig. 1) [22]. With extensive efforts devoted to the investigation of artificial enzymes and nanotechnology, creative breakthroughs have been made steadily on the catalytic mechanisms and intrinsic properties of nanozymes, as well as the application field. In the past two years, the investigation on single-atom nanozyme (SAN) has aroused numerous attention due to their outstanding activity and selectivity [23, 24]. In the research of Kim et al. [25], the Fe–N–rGO SAN showed the best catalytic efficiency for different substrates among various classical POD mimics and natural HRP. Niu et al. [26] reported that the Fe–N–C SAN not only possessed excellent enzymatic activities, but also exerted splendid stability and robustness within a broad temperature and pH range.

Since nanozymes are recognized as a class of functional nanomaterials, they possess both the unique nature of nanomaterials and enzyme-like activity [27]. The surfaces of metal and metal oxide nanomaterials are covered with a large amount of charge, which was responsible for their superb electron properties [28]. Consequently, metal- and metal oxide-based nanozymes stand out in the area of electrocatalysis, sensing and fuel cells [29, 30]. Furthermore, as promising alternatives for natural biocatalysts, they commonly retained better stability and robustness under extreme conditions than natural enzymes [5]. The prominent physicochemical properties (e.g., high surface energy, superior optical, and photothermal conversion properties), as well as simplicity in preparation and storage also broaden their applications [31]. Interestingly, the catalytic performance and physicochemical properties of metal and metal oxide nanomaterials could be easily regulated according to the practical demand [32, 33]. For instance, surface modification has been revealed as a promising strategy to increase the biocompatibility of these nanozymes [34–36]. The structure design associated with the catalytic efficiency is flexible through suitable control of synthetic conditions [37]. Given the above ascendency, the research fields of metal- and metal oxide-related nanozymes have gradually extended from environment to chemical industry, food, agriculture, biomedicine, medicine, and so forth [38–40]. Even though tremendous efforts were devoted, further promotion of this kind of nanozymes is still facing difficulties. For example, the enhancement of catalytic activity and selectivity, closely associated with the sensitivity and specificity of nanozyme-based biosensors, remains a challenge [25, 41]. In addition, the strengthened physiological stability and biological safety is vital for the spread application of nanozymes in clinical medicine [42]. Therefore, novel nanozymes and biotechnology are urgently needed to make up these defects.

Dozens of excellent reviews concerned with nanozymes have been published in recent years. Some of the reviews involved the research progress of nanozymes in a particular field [5, 21, 27, 43, 44]. Some researchers organized and revealed the natural activities and working mechanisms of specific nanozymes [45–49]. In 2019, Huang et al. [50] systematically discussed the classification, intrinsic nature, enzymatic mechanisms and potential applications of nanozymes for the first time. However, a thorough overview for metal- and metal oxide-based nanozymes is still lacking. In this review, we firstly elucidate the characteristics and synthetic methods of metal- and metal oxide-based nanozymes. Then, we will dig into the catalytic mechanisms and property regulation of these nanozymes. After introducing their appliance in biological analysis, relieving inflammation, antibacterial and cancer therapy, we finally discuss the present challenges and give a future perspective for the research of nanozymes constituted of metal and metal oxide.

## Preparing Diverse Nanozymes with Constructive Feature

Generally speaking, the existing metal- and metal oxide-based nanozymes can be roughly assorted into monometal [51], metal alloy [52–54], metal oxide [6, 55, 56], metallic core/shell nanostructure [57–59], and hybrid [60] nanomaterials in terms of constructive feature. Monometal nanozymes are usually noble metal nanomaterials possessing prominent chemical stability under natural conditions. They commonly possess facile conjugation sites to diverse biomolecule ligands and antibodies, remarkable surface plasmon resonance (SPR) properties, superior optical, and photothermal conversion properties [61–63]. However, bare monometal nanoparticles (e.g., Ag, Pt) tend to aggregate into nanoclusters, resulting in decreasing of catalytic activity [64]. What is worse, most bare noble metal nanozymes (except Au) have biological toxicity, thus limiting their application in clinical medicine. The structure, size, and morphology have been proved to influence the catalytic properties of these nanozymes [65–67]. Monometal nanozymes could be prepared through preformed-seed-mediated growth [68], high-temperature reduction method [2, 69–71], electrochemical synthesis, photochemical method, biosynthesis [72, 73], and spatially confined medium/template approach [74]. With different methods, various forms of noble metal nanomaterials (e.g., nanoparticles [14, 15], nanoclusters [10], nanorods [75], nanosheets [76], nanocubes) could be obtained. The preformed‐seed‐mediated growth is feasible for size control by changing the concentration and nature of seeds in the growth solution [77]. A variety of small molecules (e.g., tannic acid [71], citrate [78]) and macromolecular templates including DNA [79], dendrimers [80], and proteins (e.g., bovine serum albumin, human serum albumin, lactoferrin, pepsin, insulin) [2, 70] have been employed for monometal nanozymes synthesis via the high‐temperature reduction procedure. The electrochemical strategy could modulate the size and morphology of noble metal nanomaterials through controlling electrodeposition parameters during the deposition process.

Metal alloy nanozymes, containing bimetal alloys and multimetallic alloys, could be obtained via common chemical synthesis such as the one-pot strategy [81], galvanic replacement reaction [82, 83], co-reduction method [84, 85], hydrothermal growth [86], and electrodeposition method [87, 88]. Besides, biological strategy [89] and bimetallic nanomaterials printing [90] have been present as favorable synthesis method as well. The biological strategy is widely known as a green synthesis method with biological elements as the reducing agents or growth template (e.g., leaf extract, plant extract, DNA) [91, 92]. Along with the preparation of diverse nanoalloys, researchers found that the composition as well as structure affected the enzymatic characteristic of metal alloy nanozymes [93, 94]. Therefore, adjusting the proportion of various metals, enlarging porosity and specific surface area of alloy nanomaterials have been recognized as effective approaches to regulate activity. Generally, the cost of metal alloy nanozymes is much lower than that of monometal nanomaterials as the incorporation of non-precious metals. Owing to the synergistic effect of the two components, bimetal nanoalloys tend to exhibit superiorly optical and chemical properties, as well as better catalytic performance compared with noble metal nanomaterials [95]. Furthermore, the introduction of magnetic metal (e.g., Co, Fe, and Ni) could endow alloys with magnetism besides optimizing their enzymatic properties [83, 84].

Possessing high surface energy and surface-to-volume ratio, metal oxide nanozymes have been considered as promising artificial enzymes for decades [96]. The most common metal oxide nanozymes like CeO_2_, Fe_2_O_3_, Fe_3_O_4_, Co_3_O_4_, Mn_2_O_3_, and Mn_3_O_4_ nanomaterials have all been reported to possess multi-enzyme-like activities [97]. In addition, they exhibit plenty of unique properties such as magnetic, fluorescence quenching and dielectric properties [98]. Compared with precious metal nanomaterials, metal oxide nanozymes commonly exert lower price and concise synthesis process [99]. Furthermore, the low biological toxicity and favorable accumulation in biological tissues have broadened their application toward biopharmaceutical [100]. Nevertheless, there are some disadvantages of unmodified metal oxide nanozymes in terms of biology. For instance, they might show awful stability and accelerate the generation of harmful free radicals under physiological conditions [101]. Additionally, the improper surface ligands coating would lead to the failure control of drug release [102]. In recent, diverse methods have been employed for metal oxide nanozymes preparation, including the hydrothermal [103, 104], solvothermal [105, 106], pulsed laser ablation [107], co-precipitation [108, 109], sol–gel [110], and thermal decomposition method [111].

The metallic core/shell (inorganic/inorganic) nanostructure-based nanozymes could be prepared through the hydrothermal reaction [112], solvothermal method [113], sol–gel approach [114], and atomic layer deposition [115]. By combining different materials and modifying structure, researchers could regulate the stability and functionality of core/shell structure-based nanozymes conveniently [116]. For example, the introduction of SiO_2_ as coating significantly realized good stability and reduced bulk conductivity of the core particles [117]. The dispersion and biological safety of magnetite NPs encapsulated by silica could also be improved when existed in physiological environment [118]. In addition, the Au-coated nanostructure-based nanozymes have demonstrated to show enhanced chemical stability, biocompatibility, and optical properties [119, 120]. However, the accessibility between substrate and the active phase of nanozymes could be affected by coating materials [121]. Therefore, regulating coatings’ thickness, porosity, and synthesis procedure was demanded to modulate enzyme-like capacity and other chemical properties of nanozymes.

The metal- and metal oxide-based hybrid nanozymes could be prepared with organic molecules or polymers modified on the surface of metal or metal oxide nanomaterials [122, 123]. The modifications on the surface of hybrid nanozymes are used to optimize the catalytic performance, instead of acting as stabilizer during the synthesis process [124]. Generally, the intrinsic properties of hybrid nanozymes might be ascribed to size, content, and components structure [125, 126]. For instance, polymer/metal nanozymes have been revealed to show stable catalytic capacity in which metal nanoparticles are evenly dispersed in polymer [127, 128]. In parallel with enhancing catalytic activity and selectivity, the incorporation of polymer or organic molecule endows hybrid nanozymes with amazing physical, chemical, and mechanical properties (e.g., adsorption [129], water solubility [130], biodegradability [131]), thereby expanding their application in miscellaneous fields [124].

The catalytic activities and efficiency of metal- and metal oxide-based nanozymes involved in the recent reports are listed in Table 1. These nanozymes mainly imitate four kinds of natural enzymes, namely POD, oxidase (OXD), catalase (CAT), and superoxide dismutase (SOD). The Michaelis–Menten constant (*K*_m_) and maximal velocity (*V*_max_) reflects the enzyme affinity with its substrate and maximal reaction velocity respectively [132]. And the *K*_cat_ is the maximum number of substrate molecules converted to product per enzyme molecule per second. The lower value of *K*_m_ and the higher value of *V*_max_ indicate the stronger catalytic activity of nanozymes.

**Table 1:** Intrinsic activity and catalytic efficiency of typical metal- and metal oxide-based nanozymes

Nanomaterial	Surface modification	Activity	Catalyst efficiency: *k*_cat_ (s^−1^), substrate, *K*_m_ (mM), *V*_max_ (μM s^−1^)	References
Monometal				
Au NPs		GOx	18.52, glucose, 6.97, 0.63	[133]
Au NCs	Amine-terminated PAMAM dendrimer	POD,CAT,SOD	–, H_2_O_2_,16.0,0.452 (CAT)	[80]
Pt NPs	BSA	POD	–, TMB, 0.119, 0.21	[134]
			–, H_2_O_2_, 41.8, 0.167	
Pt NCs		POD	–, TMB, 0.096, 0.1414	[135]
			–, H_2_O_2_, 3.07, 0.1817	
Pd NPs	Carboxylated chitosan	POD	–, TMB,0.09, 0.177	[136]
			–, H_2_O_2_, 537.71, 0.112	
Ru NPs		HRP, OXD	–, TMB,0.234, 0.0825 (HRP)	[137]
			–, H_2_O_2_, 2.206, 0.583 (HRP)	
Cu NCs		POD	–, TMB, 0.648, 0.0596	[138]
			–, H_2_O_2_, 29.16, 0.0422	
Os NPs	Citrate	POD	1.72 × 10^3^,TMB, 0.096, 0.412	[139]
			2.35 × 10^3^, H_2_O_2_, 3.88, 0.565	
Ir NPs	Citrate	POD,CAT,OXD	5 × 10^2^, TMB, 0.0906, 1.7 (POD)	[140]
			4.4 × 10^2^, H_2_O_2_, 0.27, 1.5 (POD)	
			–, H_2_O_2_, 21.09, – (CAT)	
Rh NPs	Citrate	POD	3.87 × 10^2^, TMB, 0.198, 0.0678	[141]
			1.38 × 10^3^, H_2_O_2_, 0.38, 0.241	
Metal alloy				
Au_2_Pt		CAT	–, H_2_O_2_, 7.7066, 0.9018	[142]
AgPt NPs	BSA	CAT,POD	0.751 × 10^3^,OPD,0.129,89.71 (POD)	[17]
			1.075 × 10^3^, H_2_O_2_,76.05, 128.49 (POD)	
			183.735 × 10^3^, H_2_O_2_,54.30, 16.2 (CAT)	
Au–Pt NCs	Guanosine monophosphate (GMP)	OXD	–, TMB, 6.805, 2.538	[143]
			–, ABTS, 0.1321,0.1798	
Fe–Pt NPs		OXD	–, TMB, 0.030, 0.0142	[144]
Pd/Pt NWs		OXD	–, TMB, 0.058, 0.114	[33]
NiPd NPs		CAT,POD,OXD	–, TMB,0.11, 0.0152 (POD)	[83]
			–, H_2_O_2_, 0.66, 0.2618 (POD)	
Metal oxide				
MnO_2_ NSs	HSA	OXD	–, TMB, 0.042,0.212	[145]
Mn_3_O_4_ NPs		OXD	–, TMB, 0.08, 0.4817	[146]
Fe_3_O_4_	histidine	POD	1.8256 × 10^5^, TMB, 6.22, 0.157	[105]
			1.6965 × 10^5^, H_2_O_2_, 10.58, 0.1459	
CeO_2_ NPs		Phosphatase	–, pNPP, 0.74, 7.33 × 10^–6^	[147]
CeO_2_ NRs	SO_4_^2−^	OXD	16.55, TMB, 0.22, 0.48	[148]
Co_3_O_4_ NPs		CAT	1.63 × 10^4^, H_2_O_2_, 34.3, 11.2	[103]
Co_3_O_4_ NPs		OXD	–, TMB, 0.051, 0.033	[104]
			–, ABTS, 0.037,0.032	
Co_3_O_4_ nanoflowers		POD,CAT, OXD,SOD	–, TMB, 0.2830, 0.1052 (POD)	[149]
			–, H_2_O_2_, 5.9322, 0.0985 (POD)	
			–, H_2_O_2_,839.85, 1466.66 (CAT)	
			–, TMB, 0.0469, 0.0459 (OXD)	
NiO nanoflowers		SOD	2.6 × 10^10^, O_2_˙^−^, 0.043,35	[106]
Core/shell nanostructure				
Au@Pt		POD	1.475 × 10^3^,TMB, 0.00243, 0.04425	[150]
			2.004 × 10^3^, H_2_O_2_, 0.00407, 0.06013	
Fe_3_O_4_@MoS_2_		POD	–, TMB,0.25, 0.111	[151]
			–, H_2_O_2_, 1.39, 1.63	
Fe_3_O_4_@C NWs		POD	–, TMB,0.20, 0.0134	[152]
			–, H_2_O_2_, 0.23,0.0241	
Co@Fe_3_O_4_		POD	–, TMB,1.17, 0.379	[153]
			–, H_2_O_2_, 0.19,0.715	
Au/CeO_2_ NPs		POD,CAT,SOD	–, TMB, 0.29, 0.039 (POD)	[154]
			–, H_2_O_2_, 44.69, 0.0223 (POD)	
Cu_2_O@TiO_2_ NWs		OXD	15.25, glucose, –, 0.915	[155]
Pd cube@CeO_2_ NPs		OXD	–, TMB, 0.21, –	[156]
Hybrid nanozymes				
PVP/IrPt NPs		CAT,POD,OXD	–, TMB, 0.16, 2.25 (POD)	[157]
			–, H_2_O_2_, 0.75, 2.66 (POD)	
Fe_3_O_4_/CoFe-LDH		POD	–, TMB, 0.395, –	[158]
			–, H_2_O_2_, 10.24, –	
Co_3_O_4_@β-cyclodextrin NPs		POD	–, TMB, 0.17, 0.0281	[36]
			–, H_2_O_2_, 1.42, 0.0285	
HS-Pt NPs		OXD	–, TMB, 0.01012, –	[159]
His@AuNCs/RGO		OXD	–, TMB, 0.031, 0.0655	[160]

*BSA* bovine serum albumin, *PVP* polyvinylpyrrolidone, *PNPP p*-nitrophenyl phosphate, *LDH* layered double hydroxides, *HS* heparin sodium, *RGO* reduced graphene oxide, *His* histidine, *GOx* glucose oxidase, *HRP* horseradish peroxidase, *OPD* o-phenylenediamine, *NSs* nanosheets, *HSA* human serum albumin

## Properties of Metal- and Metal Oxide-Based Nanozymes

### Catalytic Mechanism

#### Catalase-Like Activity

CAT is a kind of binding enzyme with iron porphyrin as its prosthetic group [161]. CAT presents in the living tissues could catalyze hydrogen peroxide (H_2_O_2_) into oxygen and water, hence protecting tissues from excessive H_2_O_2_ [162]. Up to now, a series of metal-associated nanozymes, such as platinum (Pt) [51], gold (Au) [163], CeO_2_ [164], Mn_3_O_4_ [19], have been demonstrated to show CAT-like activity. Although promising in anti-inflammatory, tumor treatment, biological detection and many other fields, considerable CAT mimics still constrained by the obscure mechanism [165, 166]. Li et al. [167] verified that the pre-adsorbed OH group on the surface of noble metal served as the active site for CAT-like catalytic reaction. Although most reported nanomaterial-based CAT mimics showed favorable catalysis ability in neutral and alkaline environment, Liu et al. [80] firstly reported that amine-terminated PAMAM dendrimer encapsulated gold nanoclusters (AuNCs-NH_2_) displayed CAT-mimicking property not only in acidic environment but also over physiological pH range (i.e., pH 4.8–7.4). They speculated that the protonation of tertiary amines from dendrimers in acidic solution could stimulate pre-adsorbing OH, thus providing active sites for H_2_O_2_ decomposition to generate oxygen and water.

**Fig. 2:**
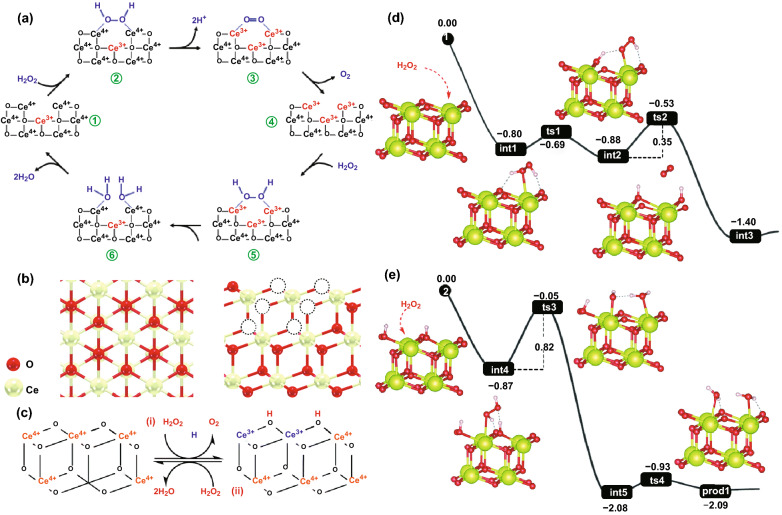
**a** Electron transfer mechanism for the CAT-mimetic activity of CeO_2_ NPs. **b** Top view (left) and side view (right) of the CeO_2_ (111) structural model. **c** Atomistic-level catalytic mechanisms for the CAT-mimicking reactions of nanoceria. **d**, **e** Energy profiles for steps (1) and (2) for CeO_2_ (111). Adapted from **a** Ref. [168], **b**–**e** [171] with permission

In terms of metal oxide nanozymes, Celardo et al. put forward a possible catalytic model of CeO_2_ NPs with CAT-mimicking properties in 2011 [168]. In the system, H_2_O_2_ was firstly bind to the 2Ce^4+^ binding site presented by the oxygen vacancy site of CeO_2_ NPs (Fig. 2a➀, ➁). Then, the fully reduced oxygen vacancy site was formed as the protons released and two electrons transferred to the two Ce^4+^ (Fig. 2a➂). The oxygen was generated from the reduced oxygen vacancy site (Fig. 2a➃). Afterwards, another H_2_O_2_ molecule was bind to the 2Ce^3+^ site (Fig. 2a➄). The homolysis of O–O bond happened with the transfer of two electrons and a uptake of two protons (Fig. 2a➅). After H_2_O molecules released, the initial Ce^4+^ sites were regenerated on nanoceria surface. Interestingly, Mu et al. reported that a larger concentration of the perhydroxyl anion (OOH^−^) contained in H_2_O_2_ molecule were existed in the neutral and alkaline solution [103]. The OOH^−^ then might interact with metal centres of Co_3_O_4_ and form the ^**·**^O_2_H due to its prominent nucleophilic ability compared with H_2_O_2_. With terephthalic acid as the fluorescent probe, it could be found that the production efficiency of the hydroxyl radical (^**·**^OH) depended on the Co_3_O_4_ concentration, indicating that the CAT-type property of Co_3_O_4_ NPs would influence the decomposition of H_2_O_2_ to ^**·**^OH. Moreover, thermodynamic and kinetic analysis revealed that there might be more “active sites” on the surface of Co_3_O_4_ NPs than natural CAT owing to the stronger affinity between H_2_O_2_ and Co_3_O_4_ compared with natural CAT.

The existing hypothetical mechanisms for the CAT-like property of CeO_2_ NPs and Co_3_O_4_ NPs mentioned above still show certain limitations due to the neglect of the real structural features discussion [169]. Therefore, Guo et al. [170] investigated the possible catalytic mechanism of CAT-type activity at atomic or molecular level, involving the base-like dissociative, acid-like dissociative, and bi-hydrogen peroxide associative mechanisms. Based on the calculation of thermochemical energies and associated activation barriers, they reported that the bi-hydrogen peroxide associative mechanism was most viable for the CAT-mimicking catalytic recycle for Co_3_O_4_. Wang et al. deeply investigated the structural and electronic properties of nanoceria to propose the atomistic-level mechanisms (Fig. 2b, c) [171]. In their model, the CeO_2_ (111) surface oxidized H_2_O_2_ molecule to form O_2_ and a reduced H_2_-CeO_2_(111) surface. Then, anotehr H_2_O_2_ molecule would react with the H_2_-CeO_2_(111) surface to produce H_2_O. As shown in Fig. 2d, the reaction between H_2_O_2_ and CeO_2_ (111) surface was exoenergetic (energy difference Δ*E* =  − 1.40 eV) with a small energy barrier (*E*_a_) of 0.35 eV. Since Δ*E* =  − 2.09 eV and *E*_a_ = 0.82 eV, the interaction between H_2_-CeO_2_(111) surface and H_2_O_2_ was also exoenergetic and kinetically favorable as well (Fig. 2e).

#### Peroxidase-Like Activity

Peroxidase, produced by microorganisms or plants, is closely related to the growth of animals and plants [172, 173]. The peroxidase family is very huge, and most peroxidases are heme enzymes with ferric protoporphyrin IX (protoheme) as the prosthetic group (e.g., horseradish peroxidase, lignin peroxidases, myeloperoxidase) [174–177]. Following the blooming exploration on enzymes, peroxidases with selenium (glutathione peroxidase, GPx), manganese (manganese peroxidase), and vanadium (bromoperoxidase) as active centers have been widely reported [178–180]. Peroxidase catalytically oxidizes organic substrates in which H_2_O_2_ acted as an electron acceptor, thereby decomposing H_2_O_2_ and effectively eliminating the toxicity of phenols and amines. In 2007, GAO et al. discovered that magnetite (Fe_3_O_4_) nanoparticles had a special property that similar to HRP [6]. Since then, a series of nanomaterials have been unraveled to serve as POD mimics, including metal materials [181], metal oxides [182], conducting polymers [183], metal organic frameworks [184], carbon nanomaterials [185], single-atom catalysts [186] and so on.

**Fig. 3:**
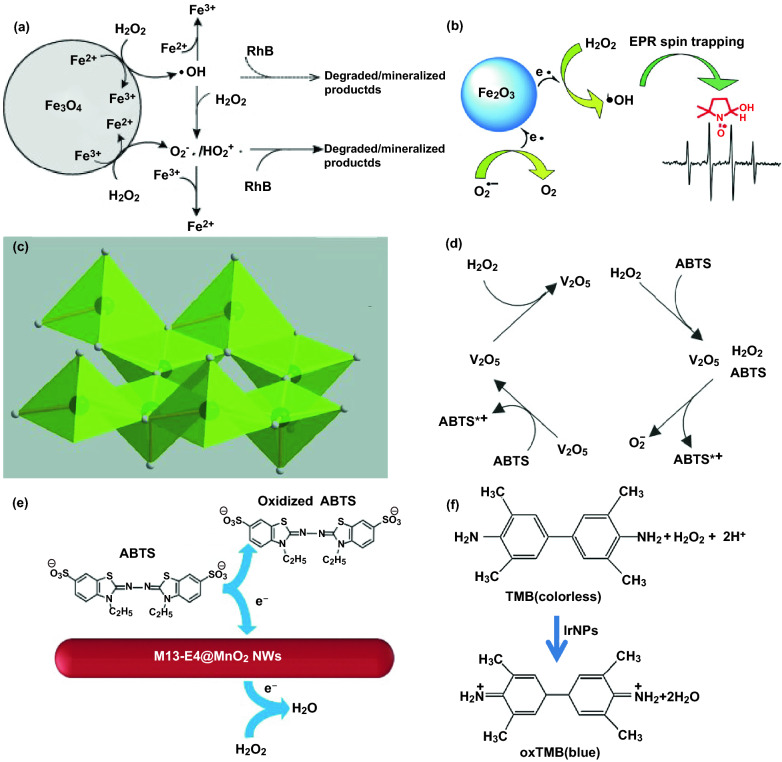
**a** Mechanism for the POD-like activity of Fe_3_O_4_ MNPs in the degradation of organic pollutants. **b** Mechanism mediated by γ-Fe_2_O_3_ NPs. **c** View of Single layer from the V_2_O_5_ structure. **d** Possible mechanism for the catalytic reaction of the V_2_O_5_ NWs. **e** Catalytic mechanism of M13-E4@MnO_2_ NWs with POD-type properties. **f** Corresponding reaction equation of TMB oxidized by H_2_O_2_ with the Ir NPs as POD mimics. *ABTS* (2,2'-azino-bis(3-ethylbenzothiazoline-6-sulfonic acid)). Adapted from **a** Ref. [187], **b** Ref. [188], **c**, **d** Ref. [189], **e** Ref. [9], **f** Ref. [71] with permission

The catalytic mechanisms of various nanomaterial-based POD mimics could generally be concluded as Fenton or Fenton-like reaction or the electron transfer process [117]. Wang et al. [187] prepared Fe_3_O_4_ magnetic nanoparticles (Fe_3_O_4_ MNPs) via a reverse co-precipitation method under ultrasonic irradiation. The possible catalytic mechanism of Fe_3_O_4_ MNPs with POD-type activity was displayed in Fig. 3a. The bound Fe^2+^ and Fe^3+^ activated H_2_O_2_ molecules that adsorbed on the surface of Fe_3_O_4_ MNPs to produce ^**·**^OH and oxygen superoxide anion (O_2_^**·**−^)/hydroperoxyl radicals (HO_2_^**·**^). Then, the ^**·**^OH and O_2_^**·**−^/HO_2_^**·**^ radicals would induce the subsequent degradation and mineralization of Rhodamine B (RhB). However, Maxim et al. [188] put forward different opinions about the generation of ^**·**^OH under conditions of the biologically relevant superoxide-driven Fenton reaction. Based on the spin-trapping electron paramagnetic resonance (EPR) experiments, they discovered that the reactions (Eqs. 1–3) at the nanoparticles’ surface rather than the metal ions released by the nanoparticles were responsible for the POD-mimicking property of γ-Fe_2_O_3_ and Fe_3_O_4_ NPs (Fig. 3b). What is more, the production effect of the catalytic centers on the surface of γ-Fe_2_O_3_ was demonstrated to be at least 50-fold higher than that of the dissolved metal ions.

1 $${\text{Fe}}^{{{\text{3}} + }} + {\text{O}}_{{\text{2}}} ^{{\cdot - }} \to ~{\text{Fe}}^{{{\text{2}} + }} + {\text{O}}_{{\text{2}}}$$

2 $$2{\text{O}}_{2} ^{{\cdot - }} + 2{\text{H}}^{ + } \to {\text{H}}_{2} {\text{O}}_{2} + {\text{O}}_{2}$$

3 $${\text{Fe}}^{{2 + }} + {\text{H}}_{2} {\text{O}}_{2} \to {\text{Fe}}^{{3 + }} + {\text{OH}}^{ - } + ^{\cdot} {\text{OH}}$$

The nanocrystalline structure of nanozymes was also considered to make contribution to the H_2_O_2_-activating ability. André et al. reported that the intrinsic POD-like activity of V_2_O_5_ nanowires was attributed to surficial properties of the nanozymes instead of free orthovanadate anions [189]. They proposed a likely reaction mechanism based on analyzing the layered V_2_O_5_ orthorhombic structure (Fig. 3c). The (001) surface and the (110) surface were predominantly connected to the selective oxidation of hydrocarbons and total oxidation, respectively. The surface sites on the exposed (010) lattice planes of V_2_O_5_ NWs was assumed to be related to their enzyme-like property. The V atoms in the (010) plane and the electron lone pairs of the bridging oxygen atoms, respectively, acted as Lewis acid and base sites. Consequently, an intermediate peroxo species was produced after the reaction between V_2_O_5_ NWs and H_2_O_2_ (Fig. 3d). Afterward, the ABTS was bind to the vanadium peroxo species via a nucleophilic attack and then oxidized into ABTS*^+^ species. The regeneration of the V_2_O_5_ NWs required another ABTS molecule since H_2_O_2_ is a two‐electron oxidant.

In recent years, the electron transfer-related mechanism was applied to a bunch of POD mimics such as IrO_2_/rGO nanocomposites [123], FePt-Au hybrid NPs [190], Co_3_O_4_ NPs [191], and AuNPs@CDs nanocomposites [122]. Han et al. [9] obtained recyclable biotemplate-based MnO_2_ nanowires with genetically engineered filamentous phages M13 as template. As illustrated in Fig. 3e, an electron transfer model was proposed for the reaction mechanism. With an electron transferred to MnO_2_ NWs, the first substrate ABTS was oxidized. Then, another electron would transfer from MnO_2_ to H_2_O_2_ and hence produced H_2_O molecules. According to the chromogenic reaction and a series of control experiments, the enhanced POD-mimetic capacity of 1D M13-E4@MnO_2_ nanozymes could be attributed to the surface effect, the small size effect and the homogeneous distribution of nanocrystals. When it comes to noble metal nanozymes, Cui et al. [71] speculated that Ir NPs could serve as the electron transfer mediators between H_2_O_2_ and 3,3′,5,5′-tetramethylbenzidine (TMB) (Fig. 3f). TMB adsorbed on the Ir surface provided lone-pair electrons from amino group to the Ir NPs, whose electron density was consequently increased. The electrons that transferred from the Ir NPs to peroxides would accelerate the oxidation of TMB and the reduction of H_2_O_2_.

#### Oxidase-Like Activity

**Fig. 4:**
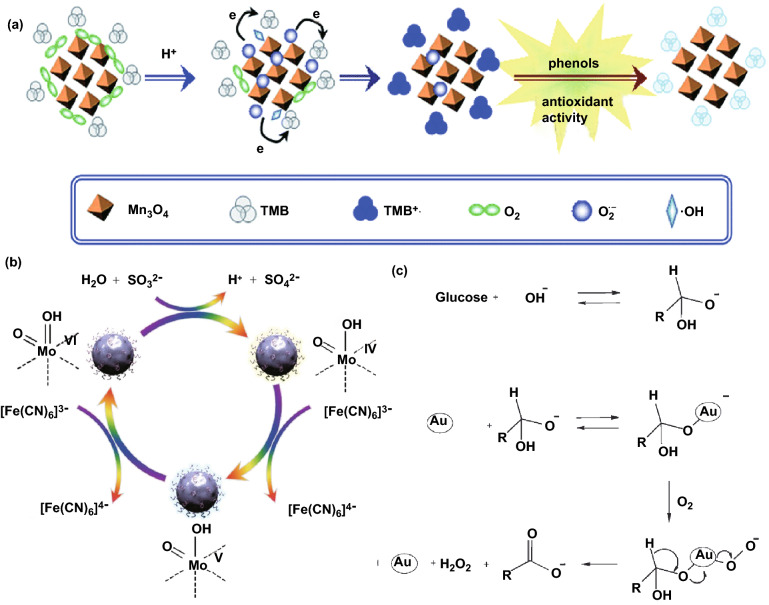
**a** Reaction of the TMB oxidized by Mn_3_O_4_ NPs with OXD-like activity. **b** Possible reaction mechanism for the SuOx-type activity of P‐MoO_3*−x*_ NPs in the presence of sulfite and K_3_[Fe (CN)_6_]. **c** Catalytic mechanism of Au NPs as GOx mimics. Adapted from **a** Ref. [196], **b** Ref. [198], **c** Ref. [199] with permission

Oxidases catalytically oxidize substrate (electron donor) and produce H_2_O or H_2_O_2_ in the presence of oxygen, which is served as the electron acceptor. The oxidase family is classified according to the acting group of donors, including amino groups, CH-OH group (GOx), Ph-OH group (polyphenol oxidase), sulfur group (sulfite oxidase, SuOx), and ferrous ions (ferroxidase and cytochrome *c* oxidase) [192]. Among them, the OXD-mimetic nanozymes that acting on amino groups were widely investigated. Up to date, a large amount of metal-based and metal oxide-based oxidase mimics have been uncovered, such as CuO [193], MnFe_2_O_4_ [194], and Pt@MnO_2_ [58]. The formation of intermediates (e.g., singlet oxygen, oxygen superoxide anion) and electron transfer process have been demonstrated to have important impacts on the OXD-type properties of these nanozymes [195]. The possible reaction mechanism of Mn_3_O_4_ NPs proposed by Zhang et al., which was illustrated in Fig. 4a [196]. The electrons that transferring from manganese to O_2_ caused the formation of O_2_^**·**−^, part of which was responsible for the generation of H_2_O_2_ and O_2_ via non-enzymatic or SOD-catalyzed dismutation. Then, some of produced H_2_O_2_ would react with the dissolved Mn^2+^ and decomposed into ^**·**^OH. Afterward, the intermediate ^**·**^OH/O_2_^**·**−^ and Mn^3+^ would oxidize the TMB, thus forming the TMB–Mn_3_O_4_ NP system. As a concerned nanomaterial, the CeO_2_ has been demonstrated to exhibit multi-enzyme-mimicking activities. Cheng et al. probed into the O_2_-dependent catalytic behavior of nanoceria and confirmed its OXD-type activity under the studied conditions [197]. In the reaction mechanism, the O_2_ molecules were adsorbed onto defect sites of nanoceria and converted into O_2_^**·**−^ under acidic conditions (Eq. 4). As the surface Ce^4+^ reduced to Ce^3+^, the TMB was oxidized into TMB_ox_ (Eq. 6). As the main intermediate, the in situ produced O_2_^**·**−^ finally regenerated Ce^4+^ via the oxidation of Ce^3+^, accompanied by the generation of water (Eq. 7). Alternatively, the oxidation of TMB could be directly initiated by O_2_^**·**−^ as well (Eq. 5).

4 $${\text{O}}_{2} + {\text{Ce}}^{{3 + }} \left( {{\text{CeO}}_{2} } \right) \to {\text{O}}_{2} ^{{\cdot - }} + {\text{Ce}}^{{4 + }} \left( {{\text{CeO}}_{2} } \right)$$

5 $${\text{O}}_{2} ^{{\cdot - }} + {\text{TMB}}_{{{\text{red}}}} \to {\text{H}}_{2} {\text{O}} + {\text{TMB}}_{{{\text{ox}}}}$$

6 $${\text{CeO}}_{2} + {\text{TMB}}_{{{\text{red}}}} \to {\text{Ce}}_{2} {\text{O}}_{3} + {\text{TMB}}_{{{\text{ox}}}}$$

7 $${\text{Ce}}_{{\text{2}}} {\text{O}}_{{\text{3}}} + {\text{O}}_{{\text{2}}} ^{{\cdot - }} + {\text{ 2H}}^{ + } \to {\text{CeO}}_{{\text{2}}} + {\text{ H}}_{{\text{2}}} {\text{O}}$$

Mechanism study on the nanozymes mimicking the other members of the oxidase family has made great progress as well. Following the exploration on the MoO_3_ NPs as SuOx mimics [198], Chen et al. synthesized PEGylated (polyethylene glycol)‐MoO_3*−x*_ nanoparticles (P‐MoO_3*−x*_ NPs) that could catalytically oxidize sulfite. As shown in Fig. 4b, the sulfite was oxidized into sulfate with the two electron oxidative hydroxylation. Following the reduction of [Fe(CN)_6_]^3−^, one electron then transferred in succession to the Mo^V^ intermediate for the stabilization of the inactive Mo^IV^ state. In terms of nanozymes with GOx-like acticity, Comotti et al. put forward a two-electron mechanism to explain the intrinsic catalytic activity of the Au NPs (Fig. 4c) [199]. In their model, the hydrated glucose anions that formed in the presence of alkali were adsorbed on the surface of AuNPs. The gold surface atoms on the hydrated glucose then activated molecular oxygen and formed the dioxogold intermediate, which provided a bridge (Au^+^–O_2_^−^ or Au^2+^–O_2_^2−^ couples) for the electron transfer. After two electrons transferring from glucose to dioxygen, the gluconic acid and H_2_O_2_ were finally generated. Zhang et al. [200] prepared crown-jewel-structured Au/Pd nanoclusters with high reactivity. The anionic charge on the top Au atoms may directly contribute to the high GOx-like activity since a hydroperoxo-like species was formed during the electron transfer progress form the anionic top Au atoms to O_2_. In addition, the PtCu NPs were reported to possess ferroxidase-like activity isolated from the impact of other ions based on the Fenton-like reaction [201]. Despite the obscure mechanism, the Pt NPs (as catechol oxidase mimics) [202], Au nanorod/ Pt nanodot structures (as ferroxidase mimics) [203], Cu_2_O NPs (as cytochrome *c* oxidase mimics) [204] and many other metal- and metal oxide-based nanozymes have broaden the way toward the prosperity of OXD mimics.

#### Superoxide Dismutase-Like Activity

**Fig. 5:**
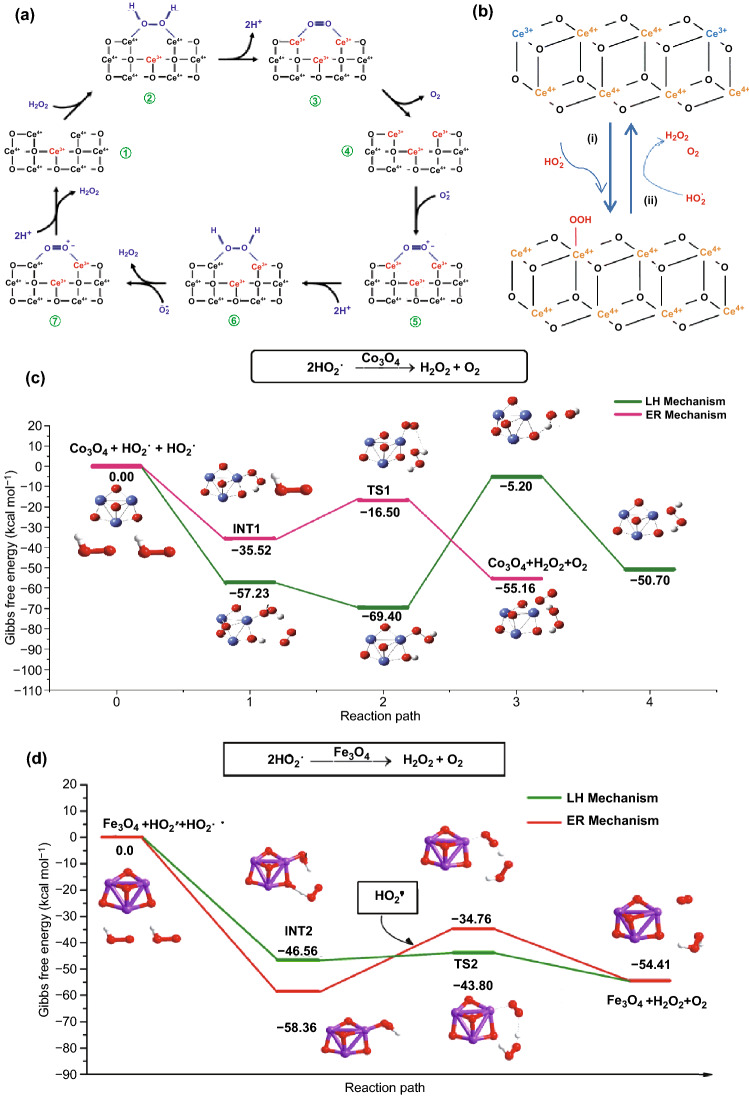
**a** Electron transfer model for the oxidation of H_2_O_2_ by nanoceria as SOD mimics. **b** Reaction mechanism for the SOD mimetic activity of nanoceria. **c**,** d** Calculated reaction energy profiles for the SOD-mimic activity of Co_3_O_4_ and Fe_3_O_4_. Adapted from **a** Ref. [168], **b** Ref. [171], **c** Ref. [170], **d** [212] with permission

Superoxide dismutase is a kind of metalloenzyme that mainly distributed in microorganisms, plants and animals. Oxidative stress, involving the increasing concentration of reactive oxygen species (ROS), is considered to be an important factor in aging and disease [205]. ROS refers to the reduction products of oxygen in the body, including oxygen radicals (e.g., O_2_^**·**−^, ^**·**^OH, HO_2_^**·**^) and certain nonradical oxidizing agents (e.g., ozone, H_2_O_2_, hypochlorous acid) [206]. SOD is selected as a favorable tool to anti-oxidation and anti-aging since it could transform superoxide anion radicals into H_2_O_2_ and O_2_ [207]. Numerous nanomaterials have been proven as SOD mimics, such as Mn_3_O_4_ [208], Au[63], MnO_2_ [209], and CeO_2_ [210]. The coupled electron-transfers model was once accepted as a rational mechanism to explain the SOD mimetic property of CeO_2_ NPs as shown in Fig. 5a [168]. Following the oxidative half-reaction (Fig. 5a➀–➃, same as that in Fig. 2a), a O_2_^**·**−^ molecule would bind to the reduced oxygen vacancy site (Fig. 5a➄). Then, H_2_O_2_ was released with the absorption of two protons and the transfer of electron from one Ce^3+^ (Fig. 5a➅). The original nanoceria state would be regenerated by repeating this reaction with a second O_2_^**·**−^ molecule (Fig. 5a➆). However, this model was questioned since Cafun et al. demonstrated the absence of spin-unpaired Ce^3+^ sites in colloidal nanoceria via means of high-energy resolution hard X-ray spectroscopy [211]. Given profound consideration about the true structure and electronic characteristics of cerium oxide, Wang et al. proposed a polished catalytic cycle mechanism for nanoceria as SOD mimics [171]. The surface defect states were critical to the enzyme-like activity in this model. After the coadsorption of HO_2_^**·**^ onto the surface of CeO_2_, the intermediate was formed as shown in Fig. 5b. Then, the reaction between the intermediate and another HO_2_^**·**^ radicals could release H_2_O_2_ and O_2_, with the nanoceria restored to the initial state.

With the assistance of rigorous density functional theory and microkinetic modeling, Guo et al. investigated the Langmuir–Hinshelwood (LH) and Eley–Rideal (ER) mechanisms to describe the SOD-like activity of Co_3_O_4_ [170] and Fe_3_O_4_ [212] respectively. As illustrated in Fig. 5c, the ER mechanism is more viable for Co_3_O_4_ as the barriers involved through ER mechanism was lower than those along LH mechanism [170]. The O_2_^**·**−^ molecule would capture a proton from water to form OH^−^ and HO_2_^**·**^. The ER mechanism began with the chemisorption of HO_2_^**·**^ on the surface of Co_3_O_4_ to generate the intermediate (INT1) and the adsorption energy was − 35.52 kcal mol^−1^. Hereafter, INT1 would react with a second HO_2_^**·**^ to release H_2_O_2_ and O_2_, accompanied by the regeneration of Co_3_O_4_. The activation barrier of the elementary reaction passing through the transition state (TS1) was 19.02 kcal mol^−1^. When it comes to Fe_3_O_4_, the LH mechanism is viable since the barrier along the LH mechanism is lower (Fig. 5d) [212]. Two HO_2_^**·**^ molecules were absorbed on the surface of Fe_3_O_4_ to from the intermediate (INT2) with OOH* and HOO* species. Then, the O–H bond of OOH* species was split and the H atom was combined with the nearby O atom of HOO* (TS2). The H_2_O_2_ and O_2_ molecule were produced with the O_2_ molecule binding to the Fe site. Finally, the H_2_O_2_ and O_2_ molecule were released.

#### Others

Compared with oxidoreductive family, the reports about metal- and metal oxide-based nanomaterials with hydrolase mimetic activities are relatively rare. The peptide-functionalized monolayer protected gold clusters (Au MPCs) have been demonstrated as mimics of nuclease, esterase and silicatein [213–216]. The functional groups present on the protecting shells of Au MPCs were fundamental to their catalytic activities [217]. In addition, the CeNPs have been uncovered to show phosphatase-like property since they could cleave the phosphate ester bond of ATP, pNPP, and *o-*phospho-l-tyrosine [218–220]. The key to their catalytic phosphate ester bond cleavage lied on the availability of cerium(III) sites. Dhall et al. prepared CeNPs with phosphatase and CAT-mimetic activities via the wet chemical method [147]. The kinetic studies using pNPP as the substrate indicated that their phosphatase-type catalytic mechanism followed the saturation-based kinetics with *V*_max_ and *K*_m_ values of 0.44 nmol min^−1^ and 0.74 mM, respectively. In their study, the tungstate and molybdate tend to inhibit the phosphatase mimetic activity of CeNPs owing to the interaction of anions with the CeNPs surface.

### Regulation of Catalytic Activity

#### Morphology

Previous studies have demonstrated that the morphology control would affect the catalytic activity of nanozymes to a large extent [146]. Exploration on the relevance between morphology and catalytic activity mainly involved surface area, pore size and volume. Tian et al. prepared VO_2_ NPs in three kinds of morphologies (fibers, sheets and rods) as POD mimics [221]. The VO_2_ nanofibers performed best in the H_2_O_2_ and glucose colorimetric assay due to their largest specific surface area. Singh et al. [222] compared Mn_3_O_4_ NPs in cube-, polyhedron-, hexagonal plates-, lakes- and flower-like morphology (Mnf). The larger size and higher surface area seemed to create higher catalytic activity of Mnf. Moreover, the multi‐enzyme property of Mnf could be ascribed to the larger pore size, which would hold the substrates and cofactor for the catalytic reactions.

**Fig. 6:**
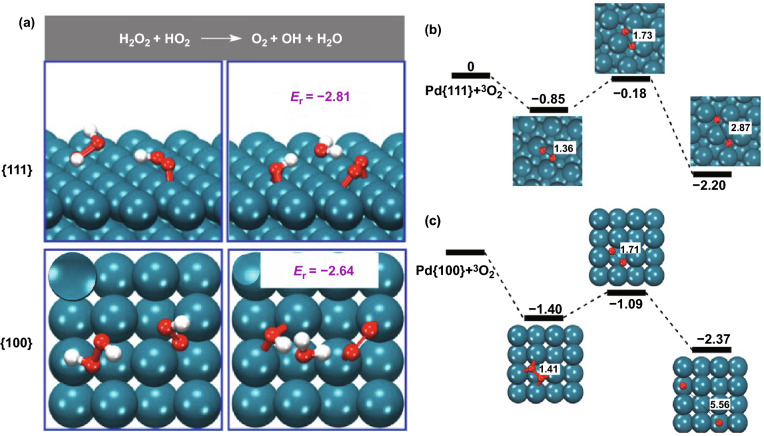
**a** Lowest-energy adsorption structures and reaction energies (in eV) for the reactions on the Pd {111} and {100} facets. **b**,** c** Relative energies (eV) of O_2_ dissociative adsorption and O–O atomic distances (Å) on the Pd {111} and {100} facets. Adapted from **a** Ref. [67], **b**, **c** Ref. [225] with permission

The effect of surface facets has gradually become a focus in morphology control as it determines surface energy or surface reactivity [223]. Huang et al. [55] found the OXD-type activity of CeO_2_ nanorods with unique {110} planes was more ingenious than that of nanopolyhedra and nanocubes. In the research of Mu et al. [224], the catalytic activities of Co_3_O_4_ materials were in the order of nanoplates > nanorods > nanocubes. The difference in lowering energy barrier and electron transfer ability might be related to distinct POD-like properties of three kinds of Co_3_O_4_ nanozymes. Ge et al. [67] reported that the Pd octahedrons enclosed by {111} facet structure showed lower surface energy, which were more sensitive to CAT-type property and ROS-eliminating capacity than the Pd nanocubes enclosed by {100} facet structure. As shown in Fig. 6a, the reaction energy on Pd {111} and Pd {100} was 2.81 and 2.64 eV respectively, indicating the more possible homolytic dissociation of H_2_O_2_ molecule on the surface of Pd {111} facet. In contrast, Fang et al. found that OXD- and POD-type activities of Pd nanocubes {100} were higher than that of Pd octahedrons {111} [225]. The binding between O_2_ and Pd {100} facet (an adsorption energy of − 1.40 eV) was much stronger than that between O_2_ and Pd {111} facet due to the higher adsorption energy at Pd {100} facet (Fig. 6b). Also, the activation energy of surficial O_2_ dissociation for {100} facets (0.31 eV) was lower than that for the {111} facets (0.67 eV). Thus, the energetically more favorable dissociative adsorption of the O_2_ molecule on the Pd {100} facet explained its higher OXD-like activity. In terms of POD capacity, the homolytic dissociation reaction on the Pd {100} facet was more feasible than on the Pd {111} facet considering the reaction energy (Fig. 6c).

#### Size

Generally speaking, size sheds significant influence on the properties of diverse nanomaterials [226]. In most cases, the nanozymes with smaller size tend to be more active in catalytic reactions ascribed to the larger specific surface area. For example, Xi et al. [32] reported the size‐dependent POD-type properties of Pd–Ir NPs within the size range from 3.3 to 13.0 nm. With an enzyme‐linked immunosorbent assay (ELISA) as a model platform, they attributed the higher catalytic properties of the smaller nanoparticles to their diffusivities and reduced steric effect. Luo et al. considered that the amount of surficial Au atoms was the key point to control the catalytic reaction rate, thus explaining the size-related GOx mimics activities of AuNPs [133]. They prepared CeO_2_ NPs with SOD- and CAT-mimetic capacities in four kinds of sizes (4.5, 7.8, 23, and 28 nm) [227]. The decreased particle sizes could increase the Ce^3+^ fraction along with enhancing catalytic efficiency. Interestingly, Liu et al. [228] discovered that the catalytic activity of β-Casein–AuNPs (β-casein functionalized AuNPs) was increased in the order of 4.2, 2.8, and 8.7 nm. Obviously, the smallest β-Casein–AuNPs did not possess the best POD-like activity. They deduced that the coated protein might affect the proximity between substrates and the nanozyme core, which also determined the enzyme-like property.

#### Surface Valence State

The controls of the surface valence state and oxygen vacancies are considered as essential factors to modulate catalytic properties. Researches have demonstrated that the surface oxidation state of nanoceria played a considerable role in tuning the enzyme-like properties of CeO_2_ due to the association between Ce^3+^ and oxygen vacancies. Pirmohamed et al. verified that the H_2_O_2_ decomposition rate of nanoceria increased with the decreasing of Ce^3+^/Ce^4+^ redox state ratios [229]. In contrast, the reduced Ce^3+^/Ce^4+^ ratio was responsible for the decay of SOD mimetic capacity [230]. Besides CeO_2_ nanozymes, Wang et al. reported that the POD mimicking activity in Ni-based nanozymes was associated with the oxidation state of Ni [231]. In their study, the catalytic performance of porous LaNiO_3_ perovskite was about 58- and 22-fold higher than that of NiO and Ni NPs, indicating the Ni oxidation state-dependent POD-like properties of Ni-based nanomaterials. Moreover, they proved the significance of Ni^3+^ in regulating catalytic activities via the comparison between LaNiO_3_-H_2_ and LaNiO_3_ nanocubes, in which the ratios of Ni^3+^ were different. With tuning copper states from Cu^0^ to Cu^2+^, Xi et al. found that the multi-enzyme-like activities (POD, CAT and SOD) of copper/carbon nanozymes were closely related to the Cu state [232]. Fan et al. realized surface valence state control on Au-based nanozymes for the first time [233]. In their system, the catalytic efficiency for substrate oxidation (TMB and H_2_O_2_) decreased with the reduced ratio of Au(I) complex in Au Aerogels.

#### Composition

The composition control of nanozymes provides possibility to tune their catalytic activity [33]. Some studies demonstrated that the catalytic performance and Raman scattering (SERS) activities of AgAu, AgPd, and AgPt NPs are more obvious than that of Ag NPs [234–236]. Similarly, alloying with other metals (e.g., Pd, Au, Cu, and Co) has also been regarded as feasible solutions to catalytic ability regulation of Pt NPs [237]. In fact, adjusting the proportion of components and designing metallic core/shell structure-based nanomaterials are both feasible solutions modulate the enzyme-like properties [154, 238]. Liu et al. speculated that the Pt/Ru molar ratio would affect electronic variation and electronic charge transfer effects of PtRu nanoalloy, thereby tuning their POD- and OXD-like activity [239]. In their work, the enzyme-type property was enhanced in the order of Pt_40_Ru_60_, Pt, Pt_75_Ru_25_, and Pt_90_Ru_10_. He et al. reported that the change of Au/Pt molar ratio not only influenced structure of AuPt alloy NPs, but also improved the catalytic reaction rates when increasing Pt/Au ratio [85]. To investigate the metallic core/shell structure-based nanomaterials, Xia et al. adjusted the amount of Ir precursor to obtain Pd–Ir cubes with different Ir shells [240]. In this work, the Ir shells at certain thicknesses would effectively increase the surface reactivity of Pd and reduce the dissociation difficulty of H_2_O_2_ molecules. Moreover, the thickness of Ir shells could enhance or weaken the ligand effect stemming from the interaction of Ir monolayer with Pd substrate, in which the Pd(100) surface with single Ir layer was more active than that with three Ir layers during the oxidation process of TMB.

Owing to the synergetic effects between ceria and heteroatoms, doping CeO_2_ with suitable foreign atoms is favorable to boost the catalytic activity [241]. By replacing Ce^4+^ ion in the CeO_2_ lattice, the incorporation of heteroatoms tends to strengthen surface defects in the CeO_2_ lattice via generating more oxygen vacancies for oxygen migration and diffusion [242, 243]. Among diverse heteroatoms, the introduction of one-dimensional nanowires achieved the best catalytic activity enhancement effect [244]. Zhang et al. synthetized CeO_2_ nanozymes doped with different metal elements (such as Ag, Cr, Co, Rh, Pd, Mn, and Ni) and possessed multi-enzyme-like activities, herein the Cr/CeO_2_ nanozymes owned best catalytic performance.The Cr^3+^ incorporation could improve surficial Ce^3+^/Ce^4+^ ratio, thus reinforcing the catalytic capacity of CeO_2_ NPs [245]. In addition to the types of doped atoms, the amounts are critical to regulate activity of nanozymes as well. Jampaiah et al. revealed that the catalytic efficiency toward TMB oxidation of 6% Fe^3+^-doped CeO_2_ NRs was the best among the CeO_2_ NRs incorporated with 3, 6, 9, and 12% Fe respectively [246]. The Raman and X-ray photoelectron spectroscopy (XPS) results indicated the higher amount of surface defects including Ce^3+^ ions and oxygen vacancies in the 6% Fe^3+^-doped CeO_2_ nanozymes.

#### Surface Modification

Surface modification ranging from functional group, inorganic ions and small molecules to macromolecules has been revealed as a promising strategy to regulate the mimetic enzyme properties of metal- and metal oxide-based nanozymes by affecting their surface chemistry [247–249]. For instance, ligands such as glutathione (GSH), dendrimer, DNA, and protein tend to protect metal nanoclusters from aggregation, thence reinforcing the stability, biocompatibility and catalytic activity of nanozymes [250, 251]. Liu et al. reported that the catalytic efficiency of the DNA-capped iron oxide NPs as POD mimics was about tenfold higher than that of naked NPs [252]. The DNA coatings not only strengthened combining capacity with the amino groups of TMB via hydrogen bonding, but also provided the *π*–*π* stacking for nucleobase interacting with the benzene rings of TMB, which effectively enhanced the affinity of Fe_3_O_4_ NPs toward TMB. Huo et al. modified Co_3_O_4_ nanoplates with the amino group (NH_2_-Co_3_O_4_), carboxyl group (COOH-Co_3_O_4_), hydroxyl group (OH-Co_3_O_4_), and sulfhydryl group (SH-Co_3_O_4_) in respective, and then systematically studied their catalytic activities [253]. Except hydroxyl group, the other functional groups all possessed positive effect to enhance POD-like activities, and among which the NH_2_-Co_3_O_4_ nanoplates ranked the first. Huo et al. considered the functional groups’ influence on the electron transfer ability of nanozymes was critical to modulating their catalytic properties. Yue et al. [254] prepared functionalized ceria nanorods catalysts M/CeO_2_ (M = Fe^3+^, Co^2+^, Mn^2+^, Ni^2+^, Cu^2+^, Zn^2+^) via chelating metal ions onto ceria nanorods CeO_2_ surface. These metal-chelated nanocerias have all possessed enhanced POD-mimicking property and Mn(II)/CeO_2_ showed best catalytic performance. The researchers found that the synergistic effect of metal ions and CeO_2_, along with the carboxyl groups served as substrate binding sites, was critical to the promotional effect on the enzymatic activity. The addition of F^−^ into nanoceria obviously caused the generation of more oxygen vacancies, facilitating electron transfer between the Ce^4+^/Ce^3+^ redox couple as well as the stimulating product desorption, thereby enhancing OXD-mimetic capacity of nanoceria by fluoride capping [255].

#### External Triggers

1. pH and temperature

Up to date, the enzyme-like activities of numerous metal- and metal oxide-based nanozymes have been verified to be sensitive to pH and temperature [17, 256–258]. The POD-type property of Fe@PCN-224 NPs was optimal in pH 3.5 with the temperature of 45 °C [259]. And the activity could remain 80% and 90% of the highest activity at 25 and 37 °C, respectively. Although an increasing number of novel nanomaterials have shown high enzyme-like property within a wide temperature range, the catalytic activity of nanozymes would slightly decrease when the temperature was not at optimal [260]. Liu et al. [261] found that the ROS eliminating activity of Pt NPs was strengthened with the increment of environment pH by the assistance of electron spin resonance (ESR) spectroscopy and spin traps. It has been reported that Pt NPs [261], Ag NPs [262] functioned as POD mimics in acidic conditions while exhibited CAT-like activities in neutral and alkaline environment. What is more, Pt and Au NPs were demonstrated to show SOD mimetic capacity under neutral conditions [63, 261]. Li et al. [167] dug into the pH-switchable enzyme-like properties of Au, Ag, Pt, and Pd nanozymes. The adsorption of H^+^ and OH^−^ ions on the metal surface was feasible under acidic and basic conditions, respectively. The base-like decompositions of H_2_O_2_ in low-pH conditions was fundamental to the POD-like activities of Au, Ag, Pt and Pd nanozymes while their CAT-type activity was related to the acid-like decompositions of H_2_O_2_ in high-pH conditions.

(2) Hydronium

The catalytic activity of nanozymes could also be affected by metal ions (e.g., Fe^3+^, Hg^2+^, Ni^2+^, Cd^2+^, and Al^3+^) and anions (e.g., S^2–^, F^–^, Cl^–^, Br^–^, and I^–^) [136, 263, 264]. For example, heavy metal ions might inhibit catalytic activities of metal- and metal oxide-based nanozymes, which could be ascribed to the metallophilic interaction between nanozymes and heavy metal ions, including the deposition of metal ions [265], the formation of alloy on the surface of nanomaterials [266], and the leaching of surface atoms [267]. The integration between heavy metal ions and the surface ligands also affected the catalytic performance of nanocomposites by deposing of ligands or decreasing affinity toward substrate [268, 269]. Han et al. conjectured that the promotional or block effects of Ca^2+^, Fe^3+^, Hg^2+^, and Mn^2+^ toward the CAT-type property of Co_3_O_4_ NPs were related to their influence on the electron transfer rate in Co_3_O_4_ [270]. In the report of Liu et al., the S^2–^ at low ion concentration tended to inhibit the POD-mimetic catalytic reactions of β-casein stabilized Pt NPs (CM–PtNPs) toward TMB while switch on their enzyme-like activity toward ABTS [264]. Besides, the sulfide-mediated activity switching efficiency decreased with the increment of S^2–^ concentration. Fluorescence spectra and X-ray photoelectron spectroscopy (XPS) data revealed that the key of S^2–^-mediated activity switching mechanism lied in the structure change of protein molecule and ratio change of Pt^2+^/Pt^0^ with the introduction of sulfide ions.

(2) Light

The photothermal effect and light-induced electron transfer have been demonstrated to be involved with the photo-enhanced enzyme-like activity of nanozymes [271–273]. With AuNPs and α-FeOOH microcrystals grown on porous carbons, Zhang et al. obtained Au/α-FeOOH–FPC catalysts with visible-light-driven enzymatic property [274]. Herein, the system temperature was raised to accelerate the process of glucose oxidation when the Au NPs converted the absorbed light energy into heat. And the generated gluconic acid could lower surrounding pH to stimulate the enzymatic reaction. Furthermore, hot electrons from plasmon-excited AuNPs promoted charge separation at the interface of Au/α-FeOOH, resulting in efficient cycling of Fe^3+^/Fe^2+^ to produce Fenton reaction. The introduction of visible light has increased the POD-type activity of Fe_2_O_3_ NPs by at least 1.2 times in the research of Zhu et al. [275]. They found that the light-related catalytic property of Fe_2_O_3_ nanozymes was concerned with the bandgap and light absorption range, which were responsible for the barrier density generation and the light energy absorption. In addition, the influence on the enzyme mimetic properties changed according to the type of light excitation. Wang et al. discovered that the catalytic activity of Au/Si/Azo (AuNPs encapsulated and dispersed by the azobenzene- modified expanded mesoporous silica) was activated under UV illumination while inhibited under visible light [276]. The control of the host–guest interaction between Azo and cyclodextrin (CD) via the isomerization between *trans* and *cis* conformations of Azo was significant to the activity regulation by UV or visible light.

(4) Others

Nucleoside triphosphates (NTPs) including adenosine triphosphate (ATP), guanosine triphosphate (GTP), cytidine triphosphate (CTP) and uridine triphosphate (UTP) have been considered as promoters for nanozymes owing to the coupling of their hydrolysis with oxidative reaction [220]. Vallabani et al. discovered that the employment of ATP could reinforce the affinity between Fe_3_O_4_ NPs and their substrate, thus maintaining the POD mimetic capacity of Fe_3_O_4_ nanozymes within a wide range of pH and temperature [277]. Interestingly, Cheng et al. [197] found that the introduction of ATP might restrain the enzymetic reaction of nanoceria in prolonged reactions despite its initial enhancing effect. They attributed the inhibition to Ce–PO_4_ complexes formation in the presence of ATP, which could interact with nanoceria and shield active centers. Furthermore, Jia et al. [278] reported that the antioxidants possessed inhibitory effect on the POD-type property of Co_3_O_4_ NPs. The addition of gallic acid (GA), tannic acid (TA) and ascorbic acid (AA) would slow the catalytic reaction toward the TMB or OPD, among which the influence of TA was the highest because of its numerous phenolic groups.

## Applications of Metal- and Metal Oxide-Based Nanozymes

### Applications in Analytical Field

As mentioned above, metal- and metal oxide-based nanozymes normally come along with unique physicochemical properties including high surface-to-volume ratio, enzymatic activity and good biocompatibility. These capabilities endow them with promising applications in target substances detection following the extensive exploration of biosensing schemes [279]. The integration of nanozymes and conventional determination technologies containing colorimetric, electrochemical, and fluorescence has gradually become optimal candidate for biological analysis. The past decade has witnessed the inclusive utilization of novel nanozyme-based sensors in detecting proteins, glucose, heavy metal ions, pathogen microorganisms and many other substances.

#### Heavy Metal Ions

**Fig. 7:**
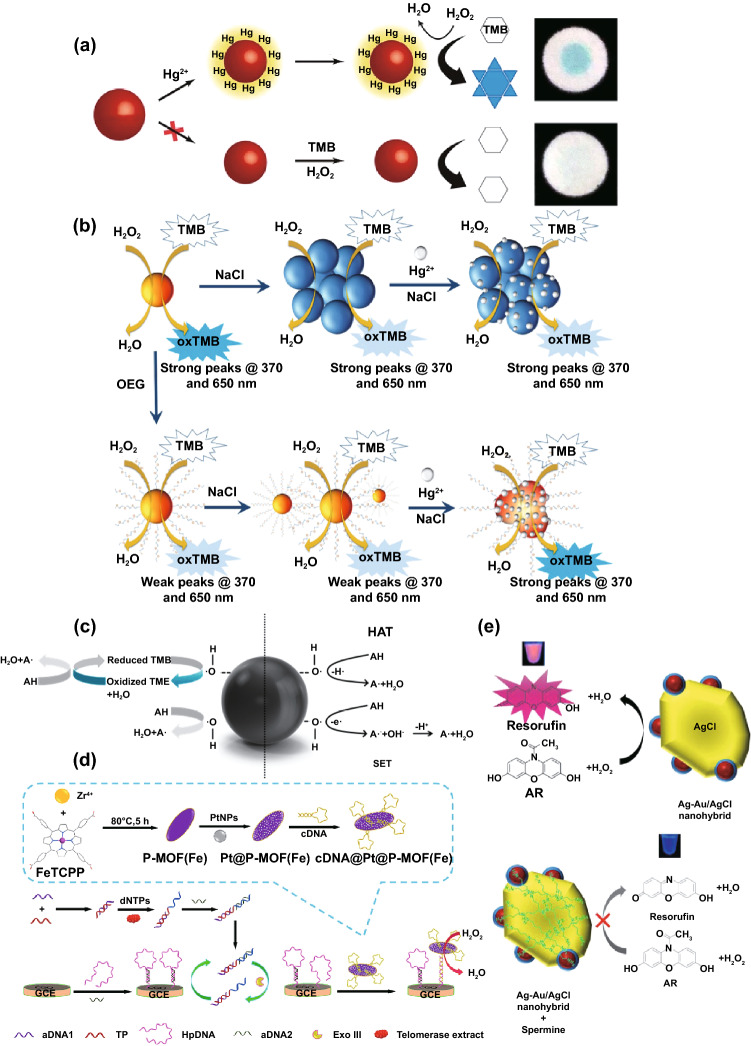
**a** Detection progress of the Hg^2+^ in distilled and tap water samples with AuNZ-PAD based on the TMB–H_2_O_2_ catalytic reaction. **b** Principle of quantitative detection of Hg^2+^ ions in seawater (3.5% NaCl) using OEG-AuNPs compared with that using bare AuNPs. **c** Detection principle of TAC based on the reaction between antioxidants and H_2_O_2_ in the presence of Pt nanozymes as POD mimics. **d** Synthesis of cDNA@Pt@P-MOF(Fe) as the signal probe for the analysis of telomerase activity. **e** Preparation of Ag-Au/AgCl nanohybrid with OXD-like and POD-like activity and the working mechanism of Spm detection. *GCE* Glassy carbon electrode, *TP* telomerase primer. Adapted from **a** Ref. [226], **b** Ref. [293]. **c** Ref. [301], **d** Ref. [302], **e** Ref. [303] with permission

Previous studies have illustrated that excessive heavy metal ions are one of the culprits of environmental pollution [280]. Furthermore, heavy metal ions could invade human body through water and food, resulting in permanent chronic poisoning [281]. Therefore, detecting heavy metal ions is of great significance to protect ecology and human health. Nevertheless, most analytical platforms (e.g., atomic absorption spectrometry, energy-dispersive X-ray, and inductively coupled plasma mass spectrometry) for heavy metal ion analysis relied on expensive instruments and professional technicians [282]. Nanozymes provided a potential to simultaneously improve the performance of metal ion detection with low cost. For instance, Han et al. designed a portable paper chip based on AuNPs (AuNZ-PAD) to investigate Hg^2+^ in distilled and tap water samples, in which Au–Hg^2+^ integration could influence enzyme-like catalytic activity of AuNPs and caused paper discoloration (Fig. 7a) [226]. This ultrasensitive AuNZ-PAD further cooperated with mobile phone camera, effectively reducing the cost of assay and simplifying the operation.

Among the classical analytical assays basing nanozymes, colorimetric stood out for the operation convenience. Some references concluded that heavy metal ions might enhance or inhibit the POD-like property of nanozymes [283–285]. Hence, histidine(His)-Pd [268], MMoO_4_ (M = Co, Ni) [286], DNA-Ag/Pt [287], MnO_2_ [288] have been synthesized for Ag^+^ [268], Cu^2+^ [286], Hg^2+^ [287, 288] monitoring by colorimetric assay. In addition, Pb^2+^ ions would accelerate the AuNPs leaching in presence of S_2_O_3_^2−^ and lead to less oxidation of TMB, expanding the Pb^2+^ determination with the assistance of nanozymes [289, 290]. Xie et al. [291] fabricated a colorimetric probe by using metallic nanozyme to determine Pb^2+^. The Au@Pt NPs served as POD mimics were introduced, which could detect Pb^2+^ ions in the lake water samples within a linear range from 20 to 800 nM.

As high electrolyte has an adverse effect on the catalytic performance and stability of nanozymes, analyzing heavy metal ions in seawater is much more difficult than other liquid samples such as lake water and drinking water [292]. Logan et al. quantitatively determined mercury ions in complicated water matrices using OEG-Au complex by functionalizing AuNPs with oligo-ethylene glycol (OEG) [293]. In this proposal, OEG-AuNPs exhibited enhanced stability and weakened catalytic properties in a wide pH range under high NaCl concentration, which effectively ameliorated the poor stability of bare-AuNPs (Fig. 7b). The Hg^2+^ detection limit of coastal seawater by this platform was 13 ppb in only 45 min.

#### Biomarkers

Biomarkers refer to biochemical indicators that mark the structure or functional changes of biosystems including organ, tissue and cell. The exploration of biomarkers is beneficial to clinical diagnosis, drug analysis and ecosystem protection. Enormous effort has been made in nanozyme-based biomarker detecting, including biological macromolecules (e.g., acid phosphatase (ACP) for prostate cancer [149]; human epidermal growth factor receptor-2 (HER2) for breast cancer [294, 295]; carcinoembryonic antigen (CEA) for rectal cancer [296, 297] and benzo[*a*]pyrene-7,8-diol 9,10-epoxide–DNA (BPDE–DNA) for woodsmoke exposure [298]) and small molecule biomarkers (e.g., sarcosine for prostate cancer [299] and uric acid [300]). Pedone et al. [301] developed a colorimetric approach to determine the total antioxidant capacity (TAC) in saliva on basis of the reaction between antioxidants and H_2_O_2_ in the presence of Pt nanozymes, which was acted as POD mimics. TAC acted as an important biological indicator closely associated with oxidative stress. It reflected the total effects of enzymes and non-enzymatic analytes in the body. The combination of Pt nanozymes and ^**·**^OH radical substrates allowed the detection scheme sensitive to both single electron transfer (SET) and hydrogen atom transfer (HAT) reactions (Fig. 7c).

The improvement in signal transduction rate is a breakthrough to raise the sensitivity of biomarker detection [298]. Thence, metal- and metal oxide-based nanozymes functioned as signal amplification has boosted biomarkers analysis in sundry assays involving electrochemical, fluorescent and so on [300]. Ling et al. obtained Pt@ P-MOF (Fe) nanozymes by growing ultra-small Pt nanoparticles on metalloporphyrin metal organic frameworks [302]. The novel artificial nanozymes were employed as signal probe, allosteric switch of DNA and Exo III recycling amplification in their electrochemical template for telomerase detection (Fig. 7d). The catalytic property of Pt NPs on P-MOF (Fe) could decompose H_2_O_2_, and hence strengthened the electrochemical signal. Kuo et al. [303] synthesized Ag-Au/AgCl nanohybrid with OXD- and POD-type capacities for spermine (Spm) analysis in urine, which could act as the diagnostic indicators for liver cancer and stroke. As is shown in Fig. 7e, Spm inhibited fluorescent molecules generation of H_2_O_2_-Amplex Red (AR) system when in the presence of Ag-Au/AgCl, thereby realizing highly selective and ingenious determination of Spm.

#### Pathogen Microorganisms

The analysis of pathogenic microorganisms, ranging from viruses, bacteria, parasites to prions, is crucial to prevention and control of infectious diseases [304]. The nanozymes have become powerful competitors for natural enzymes in field of pathogen detection due to their low-cost (especially for foodborne bacteria), timesaving operation and sensitivity [305–307]. For instance, Cheng et al. employed Pd@Pt NPs as a signal amplifier in the lateral flow immunoassay (LFIA) assays for *Salmonella Enteritidis* (*S. enteritidis*) and *Escherichia coli* (*E. coli*)* O157:H7* [57]. The integration of Pd@Pt NPs and smartphone-based device offered a portable platform for fast detection of foodborne pathogens. The studies involving nanozyme-based pathogen analysis in the past 5 years are listed in Table 2. All the metal- and metal oxide nanozymes mentioned in this table were functioned as POD mimics.

**Table 2:** Nanozyme and analysis method for pathogen microorganism detection reported in recent years

Pathogenic microorganisms		Nanozyme	Method	References
RNA virus	Avian influenza A (H5N1)	Au	Colorimetric immunoassay	[310]
	Influenza virus A (H1N1)	Au	Magnetic nanozyme-linked immunosorbent assay (MagLISA)	[311]
	Murine Norovirus (MNV)	Au	Colorimetric immunoassay	[62]
	Mumps virus	Au@Pt@mesoporous SiO_2_	Enzyme-linked immunosorbent assay (ELISA)	[64]
	Measles virus	Au@Pt	ELSA	[312]
DNA virus	Rubella virus	Au@ Pt	ELISA	[313]
Gram-positive bacteria	*Enterobacter sakazakii* (*ES*)	Fe_3_O_4_	Nanozyme strip	[314]
	*Listeria* *monocytogenes (L. monocytogenes)*	Fe_3_O_4_	Colorimetric	[315]
	*Bacillus subtilis (DH *${\infty }$*)*	Dop- Fe_3_O_4_	Colorimetric	[316]
	*Streptococcus mutans*	Fe_3_O_4_/Sm_n_(*n* = 1,2,3)	Colorimetric	[317]
	*S. aureus*	Fe_3_O_4_@SiO_2_-Pt	ELISA	[318]
		Co_3_O_4_	Magnetophoretic chromatography	[319]
		Cu-MOF	Colorimetric immunoassay	[320]
Gram-negative bacteria	*Pseudomonas aeruginosa (P. aeruginosa)*	Au	Colorimetric and electrochemical detection	[69]
	*E. coli O157:H7*	Au	Immunochromatographic Assay(ICA)	[321]
		Pd–Pt	Lateral flow assay (LFA)	[322]
		Pt-Au	ICA	[323]
		Pd@Pt	LFIA	[57]
	*S. enteritidis*	Pd@Pt	LFIA	[57]
		Fe-MOF	Colorimetric immunoassay	[324]
	*Escherichia coli (XL1)*	Dop- Fe_3_O_4_	Colorimetric	[316]

In contrast to POD mimics, other enzyme-like activities of nanozymes are waiting for further development in biological sensing. Yao et al. [308] designed a colorimetric immunoassay scheme to investigate *Staphylococcus aureus *(*S. aureus*) with the assistance of magnetic carbon dots (Mag-CDs) and AgNCs. AgNCs with OXD-mimicking properties could accelerate oxidatiing *o*-phenylenediamine (OPD) to produce yellow products. And the Mag-CDs were introduced to capture bacteria in their system. Bu et al. [309] built a point-of-care (POC) platform to analyze *Salmonella sp.* and *E. coli O157:H7* by using MnO_2_ nanoflowers with CAT-type activity. Besides, MnO_2_ possessed bacteria recognition ability via the binding between Con A and O-antigen on the bacterial surface.

#### Antibiotic

**Fig. 8:**
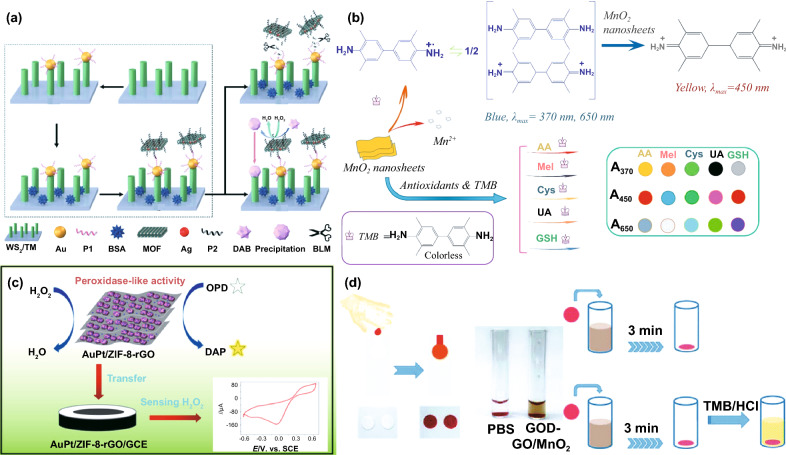
**a** Fabrication of Ag/ZnMOF-based PEC biosensor with Au/WS_2_ photoelectrode as a PEC matrix for detection of BLM. **b** Colorimetric sensor assay based on MnO_2_ nanosheets with TMB as substrates for simultaneous detection of multiple antioxidants. **c** Detection of H_2_O_2_ based on AuPt/ZIF-8–rGO as POD mimics. **d** Application of the sensor platform based on GOD-GO/MnO_2_ in blood glucose quantitative analysis. Adapted from **a** Ref. [330], **b** Ref. [340], **c** Ref. [349],** d** Ref. [362] with permission

The dose control of antibiotics, which sheds significant influence on antibacterial and anti-cancer treatment, has been a hot topic in the medical field. It has been demonstrated that overdose causes serious side effects, while insufficient antibiotics are unconducive for clinical therapy [61, 325]. While, the pioneering works of antibiotic determination, including liquid chromatography-mass spectrometry (LC–MS) [326], electrochemical [327], high performance liquid chromatography (HPLC) [328], etc. suffer from time consuming, high cost, complicated operations and poor sensitivity. The prosper of Au nanozymes with intrinsic POD-like activity provided possibility to tune the functionalization of existing methods in analyzing multiple antibiotics (e.g., doxycycline [325], kanamycin [61], tetracycline [329]). Kong et al. [330] designed a novel photo-electrochemistry (PEC) biosensor for bleomycin (BLM) detection, which was natural antibiotics for Hodgkin's disease, cervical cancer therapy. The biosensor reached a detection limit to 0.18 nM in which Ag/ZnMOF nanozymes acted as a signal amplifier and Au NPs/tungsten sulfide nanorod array (Au/WS_2_) photoelectrode used as a PEC matrix (Fig. 8a). When the Au/WS_2_ photoelectrode generated PEC signals under light, the Ag/ZnMOF nanozymes with mimetic POD properties reduced the background signal via the catalyzing reaction between H_2_O_2_ and 3,3-diaminobenzidine (DAB), thus greatly improving the sensitivity and specificity of BLM analysis.

#### Antioxidant

Antioxidants, substance to scavenge ROS or free radicals, could prevent human body from cell apoptosis and nerve damage induced by oxidative stress [331]. Nevertheless, inappropriate supplementation of antioxidants may result in diseases and increase risk of death. Therefore, quantitatively analyzing antioxidants is of great significance. The nanozyme-related antioxidant detection is based on the inhibition of antioxidants on the nanozymes’ catalytic activities [260, 278]. Following the evolvement of nanozymes and biosensing technology, the sensitive colorimetric determination for antioxidants has been extensively discussed, including ascorbic acid (AA, based on CoMn/NF@C [332], Pt/CeO_2_ [333], Fe_3_O_4_/CoFe-LDH [158], Mn-CDs [334], etc.,), GSH (based on SPB-MnO_2_ [335], Mn_3_O_4_ [336], Ir [337], V_2_O_5_ [338], etc.,) and l‑Cysteine (l‑Cys, based on Fe_3_O_4_ [339], etc.,). Most existing biosensors were designed for specific antioxidant analysis, while approaches for multiple antioxidants detection are scarce. Huang et al. [340] designed a MnO_2_ nanosheets triggered colorimetric sensor array for simultaneous discrimination of UA, GSH, AA, l‑Cys, and melatonin (Mel) in serum (Fig. 8b) [340]. The inhibitory effects on the catalytic performance of MnO_2_ nanosheets vary according to the kind of antioxidants, resulting in different degrees of TMB oxidation and generating multicolors. Since the absorbance values at 370, 650, and 450 nm would change, the corresponding absorbance values A_370_, A_450_, and A_650_ were employed as three cross-reactive sensing elements in the visual colorimetric sensor array. The detection results revealed that the sensor could precisely and rapidly identify the five antioxidants and their mixture at a low concentration.

#### Other Substances

1. **H**_**2**_**O**_**2**_

As a byproduct of respiratory metabolism, H_2_O_2_ is one of most common molecule in biological tissues [341]. When the concentration is at an abnormal status, H_2_O_2_ would cause damage to health and might induce oxidative stress related diseases [342]. Besides, hydrogen peroxide was widely used in biopharmaceuticals, environmental management, food manufacturing and some other fields due to its strong oxidant properties [343]. A bunch of methods have been designed to monitor H_2_O_2_ in various matrices considering its significant roles in biological metabolisms and broad utilization in industrial production [341, 344]. Among these assays, colorimetry and electrochemistry have gradually became main technologies for H_2_O_2_ determination owing to low cost, high sensitivity and selectivity [345]. Up to now, a variety of metal- and metal oxide-based nanozymes (e.g., CuO-g-C_3_N_4_ [346], MnO_2_ [347], V_2_O_5_-CeO_2_ [348]) have been exploited for electrochemical analysis. Zhang et al. fixed ZIF-8 on graphene oxide (ZIF-8–rGO) and further synthesized AuPt/ZIF-8–rGO with POD-like activity to practically track H_2_O_2_ in human serum samples (Fig. 8c) [349]. The AuPt/ZIF-8–rGO-based electrochemical scheme showed remarkable electroanalysis performance along with excellent sensitivity and selectivity. This work reached the detection limit of 19 nM (*S*/*N* = 3), which obtained the lowest detection limit compared with previously reported electrochemical sensors.

The color change of peroxidase substrate (e.g., TMB) triggered by hydrogen peroxide is the foundation in colorimetric detection of H_2_O_2_. Diverse POD mimics (e.g., Cu_2_O–Au [350], Fe–N–C [351], Cu(II)-coated Fe_3_O_4_ [352], PtCu [353], V_2_O_5_ [341], C-dots/Fe_3_O_4_ [130], and Rh [354, 355]) have been developed to manufacture colorimetric sensors. To our knowledge, the currently lowest detection limit of H_2_O_2_ based on colorimetry is 0.0625 µM reported by Tripathi et al. [356], and the palladium nanoclusters (Pd NCs) were designed by biological methods firstly in their study, in which Pd NCs were served as POD mimics.

(2) **Glucose**

Glucose is an indispensable nutrient for metabolism in organisms. The heat released during its oxidation reaction is a considerable energy source required by life events [357]. However, a surfeit of glucose might cause various diseases, including hyperlipidemia, arteriosclerosis, hypertension, diabetes and so on [358]. The concentration of glucose in blood or urine is a crucial indicator of physical condition [357, 359]. By combining the catalytic performance of glucose oxidase (GOD) and nanozymes with POD-type activity (e.g., Zn–CuO [331], Au@Ag [360], MoO_3_/C [331], Ag [361], and Pt [135]), numerous optical technologies have described for glucose analysis in serum[135], beverage[279], and urine [331, 361] samples. Blood pretreatment and serum extraction were often demanded in conventional blood glucose detecting programs. To simplify determination steps, Lee et al. [362] designed a protocol that could directly monitor glucose in whole blood and avert pretreatment. They prepared a GOD-conjugated graphene oxide/MnO_2_ (GOD-GO/MnO_2_) sensor platform for quantitatively analyzing blood glucose with a detection limit of 3.1 mg dL^−1^ (Fig. 8d). The results indicated that this colorimetric sensor possessed clinical potential for blood glucose monitoring of diabetic patients.

### Application in Antibacterial

**Fig. 9:**
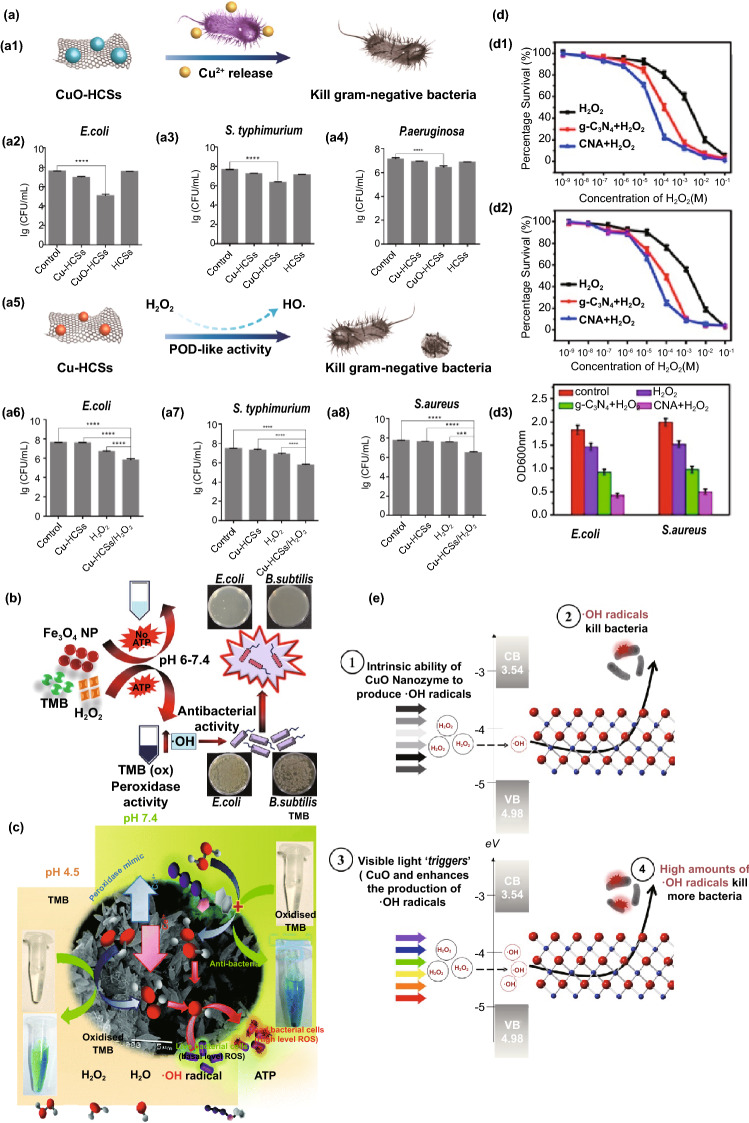
**a1**, **a5** Antibacterial mechanism of Cu/C nanozymes with two Cu states (Cu^0^ and Cu^2+^). The actual antibacterial ability of CuO-HCSs, Cu-HCSs and HCSs against **a2** *E. coli*, **a3** *S. typhimurium*, and **a4** *P. aeruginosa*. The actual antibacterial ability of Cu-HCSs, H_2_O_2_ and Cu-HCSs/H_2_O_2_ against **a6** *E. coli*, **a7** *S. typhimurium* and **a8** *S. aureus. ***b** Antibacterial activity against *E. coli* and *B. subtilis* of Fe_3_O_4_ NPs before and after ATP introduction at pH 6–7.4. **c** Catalytic activity of CeO_2_ nanocrystals before and after ATP introduction at pH 4.5 and 7.4. The bacterial viability of **d1** *E. coli* and **d2** *S. aureusand* with different treatments (H_2_O_2_, g-C_3_N_4_ + H_2_O_2_, CNA + H_2_O_2_). **d3** Optical density at 600 nm of bacterial suspension in different solutions. **e** Schematic illustration of the antibacterial principle of CuO NRs with the light as external triggers. Adapted from **a** Ref. [232], **b** Ref. [378], **c** Ref. [379], **d** Ref. [382], **e** Ref. [369] with permission

The lack of non-antibiotic therapies and multiple drug resistance caused by bacteria diseases become one of the most serious problem, which threatens human health [363–365]. In the process of developing optimal antibacterial strategies, nanometallic materials have been discovered to exert antimicrobial nature [366, 367]. In addition, POD and OXD mimics were verified to catalyze producing harmful ROS, ranging from H_2_O_2_, superoxide, hydroxyl radicals to other small reactive molecules [27]. Hence, metal- and metal oxide-based nanozymes (e.g., V_2_O_5_ [368], CuO [369], CeO_2_ [370], Au/MOF[371], and Tb_4_O_7_ [372]) have been gradually regarded as promising bactericides. For example, Fe_3_O_4_ NPs with POD-like properties could decompose H_2_O_2_ to generate toxic ^**·**^OH for bacterial infections treatment [373]. Evidence has emerged that enzyme mimic abilities of nanomaterials are closely associated with their composition and structure, which would affect antibacterial capacity [374]. Xi et al. [232] designed two types of copper/carbon nanozymes including two Cu states (Cu^0^ and Cu^2+^). The copper/carbon nanozymes displayed multi-enzyme activities and antibacterial mechanism dependent on Cu states. In the study, Xi et al. concluded that hollow carbon spheres (HCSs) modified with CuO (CuO-HCSs) nanozymes could induce Gram-negative bacteria death (*E. coli* and *P. aeruginosa*) when releasing Cu^2+^. While the key of Cu-HCSs nanozymes to resist Gram-positive (*Salmonella typhimurium*,* S. typhimurium*) and Gram-negative bacteria (*E. coli* and *P. aeruginosa*) was based on POD-type activity, which was responsible for ROS generation (Fig. 9a).

The pH-dependent catalytic activity of nanozymes has been demonstrated that would limit their antimicrobial application under neutral pH, and was beneficial to grow bacteria like Escherichia coli, Staphylococcus aureus and so on [375, 376]. Fortunately, ATP served as modulators has been reported to improve the POD-like property of nanozymes, and it could interact with iron ions to produce ^**·**^OH under neutral pH [128, 377]. Therefore, Vallabani et al. [378] employed ATP as a synergist to enhance the catalysis ability of citrate modified Fe_3_O_4_ NPs. The results showed that Fe_3_O_4_ NPs exhibited superior antibacterial performance against *E. coli* and *Bacillus subtilis* (*B. subtilis*, gram positive) in presence of H_2_O_2_ under a neutral pH environment with the assistance of ATP (Fig. 9b). Chishti et al. discovered that fluorite-structured CeO_2_ nanocrystals with ~ 23.04% Ce^3+^ had recyclable POD-like activity [379]. Mechanism investigation indicated that the reduction of substrate affinity caused by ATP is the key to improve the low enzyme-like activity of nanoyzymes in a neutral environment (pH 7.4), further strengthening the sterilization sequel against both gram-positive (*S. aureus*) and gram-negative (*E. coli*) bacteria (Fig. 9c).

Besides optimizing the catalytic capacity, applying external triggers to control their antibacterial activity is essential to develop nanozyme-based antibacterial agents. Otherwise, the sustained action of nanozymes might induce bacteria to yield drug resistance. Karim et al. firstly reported that light could act as an external spark to control nanomaterials’ catalysis [369]. A highly basic tertiary amine could produce visible light to excite CuO NRs. The increment of light intensity enhanced the affinity of CuO NRs and H_2_O_2_, thereby improving the POD-like activity and antimicrobial properties (Fig. 9e). Results showed that CuO NRs catalyzed H_2_O_2_ under visible light irradiation to output ^**·**^OH with 20 times higher than that under no light.

The exaltation of H_2_O_2_ sterilization efficiency has become an issue of increasing concern as H_2_O_2_ is a crucial and easily available ROS. Although numerous studies were devoted to this issue, applications of these systems were still restricted by the health hazard from high concentration of H_2_O_2_ (greatly higher than biologically relevant concentration) [380, 381]. Wang et al. integrated Au NPS with graphitized carbon nitride (g-C_3_N_4_) to synthesize non-toxic ultra-thin g-C_3_N_4_@AuNPs (CNA) nanozymes with high POD catalytic activity [382]. CNA nanozymes were firstly reported to possess excellent bactericidal properties under biosafety level of H_2_O_2_, and could efficiently decompose DR-biofilms to inhibit bacteria growth (Fig. 9d). In vitro experiments proved that CNA system provided significant advantages in preventing bacterial infections and accelerating wound healing.

### Application in Relieving Inflammation

**Fig. 10:**
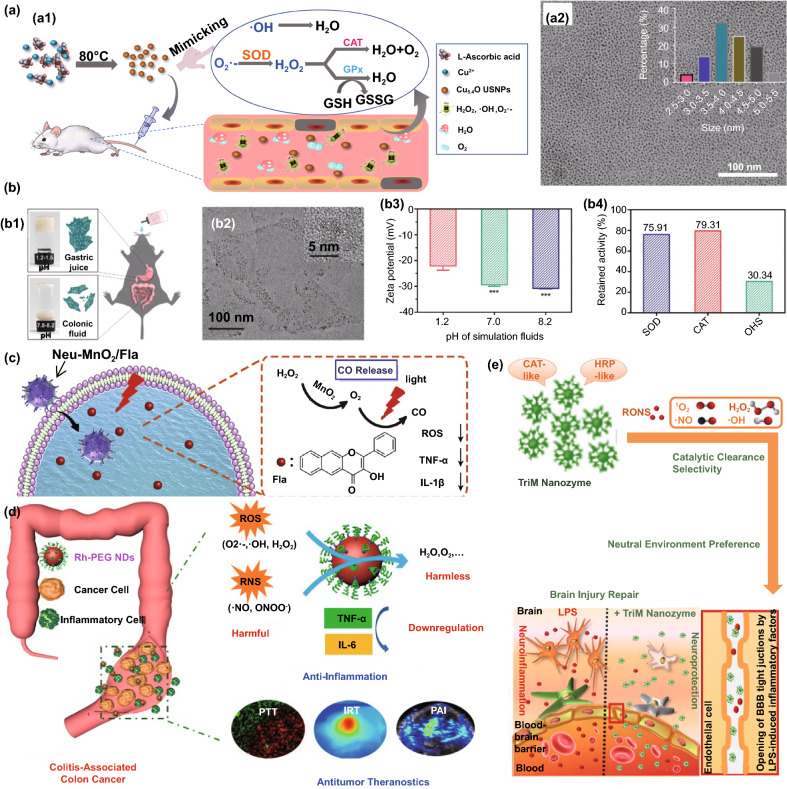
**a1** Schematic illustration of the ROS scavenging and anti-inflammation function of Cu_5.4_O USNPs with the mimic enzyme properties of CAT, SOD, GPx ability. **a2** TEM image and particle size distribution of Cu_5.4_O USNPs;** b** Stability and enzymatic activity of CeO_2_@MMT(1:9). **b1** Delivery process of CeO_2_@MMT through the simulated stomach (pH 1.2–1.5) and colon (pH 7.8–8.2) fluids via oral absorption. **b2** TEM image of CeO_2_@MMT(1:9) after treating with HCl solution (pH≈1.2) for 4 h at 37 °C. **b3** Zeta potentials of CeO_2_@MMT in simulated stomach and colon fluids. **b4** CAT- and SOD-mimicking property and ^**·**^OH scavenging activities (OHS) of CeO_2_@MMT treated with simulated gastric fluid. **c** The facilitated in situ CO release for synergistic anti-inflammatory effects induced by MnO_2_ nanozymes modified with neutrophil membrane. **d** Rh-PEG NDs with excellent RONS scavenging ability, multi-enzyme-like activity and high photothermal conversion efficiency for relieving colon inflammation and anti-tumor treatment. **e** Application of PtPdMo nanozymes with multi-enzyme-like activity and high catalytic selectivity in improving neuroinflammation. *PTT* Photothermal therapy, *PAI* Photoacoustic imaging, *IRT* interventional radiotherapy. Adapted from **a** Ref. [392], **b** Ref. [394], **c** [395] **d** Ref. [396], **e** Ref [52] with permission

Inflammation, including acute and chronic inflammation, is regarded as a precursor to certain diseases [383]. An obvious feature of inflammatory tissue is the increasing of reactive oxygen or nitrogen species (RONS) content [384, 385]. Owing to the ROS scavenging ability, favorable stability in extreme environments and excellent biocompatibility, nanozymes have been indicated to be potential substitutes for broad-spectrum antioxidants in terms of inflammation treatment [386–388]. So far, a variety of metal-based and metal oxide-based nanozymes (such as Mn_3_O_4_ [56], CeO_2_ [389], Pt/CeO_2_ [390], and Cu-TCPP MOF [391]) have been reported for anti-inflammatory therapy. The main challenge to realize clinical transformation of nanozymes is to enhance the ROS eliminating performance and simplify nanomaterials’ structure. Liu et al. synthesized ultra-small Cu_5.4_O nanoparticles (Cu_5.4_O USNPs) with mimic enzyme properties of CAT, SOD and GPx (Fig. 10a1) [392]. The ultra-micro size of Cu_5.4_O USNPs ensured their biocompatibility via the rapid removal of nanomaterials in the kidney (Fig. 10a2). Cu_5.4_O USNPs were confirmed to protect healthy cells from ROS at extremely low dosage. They also showed the promoting effect on the treatment of acute kidney injury, acute liver injury and wound healing in animal experiments. Wu et al. introduced RuO_2_-PVP NPs to set up a therapeutic nano-platform for inflammation alleviation and neuroprotection [393]. In this work, RuO_2_-PVP NPs with multi-enzymatic properties effectively protected lipid, DNA and protein from oxidative stress in parallel with the broad-spectrum ROS elimination performance against inflammation and Parkinson’s disease in vivo. Yao et al. [56] expanded the use of Mn SOD in anti-inflammatory. Their team demonstrated the multiple enzyme mimics activities of Mn_3_O_4_ NPs, which could scavenge superoxide free radicals, H_2_O_2_ and hydroxyl free radicals. In in-vitro experiments, the ROS-eliminating level of Mn_3_O_4_ NPs was much higher than traditional CeO_2_ nanozymes. The experimental results indicated the obvious prospects of Mn-based nanozymes in treating and preventing ROS-mediated neuroinflammation.

The combination of nanozymes and other kinds of anti-inflammatory agents could bring a turning point for refractory inflammatory diseases. For example, the lack of targeting strategies and the risk of side effects with increasing dosage increased the difficulty in treating inflammatory bowel disease (IBD) [397]. By growing CeO_2_ NPs in situ on montmorillonite (MMT) sheets, Zhao et al. designed CeO_2_@MMT nanozymes with SOD-type, CAT-type and ^**·**^OH scavenging properties to directly target the inflammatory colon for IBD therapy [394]. In this system, MMT alleviated the potential nanotoxicity of CeO_2_ NPs via reducing their systemic absorption, which in turn endowed MMT sheets with ROS eliminating activity. Animal experiments have also proved that the nanozyme-based drugs were suitable for oral delivery and stable in gastrointestinal environment (Fig. 10b). CeO_2_@MMT exhibited good targeting for colon disease sites, effectively treating IBD induced by dextran sulfate sodium in mice model. Although carbon monoxide (CO) gas therapy was recently revealed as a novel anti-inflammatory strategy, it still suffered from the low tissue specificity and troublesome amount control [398–400]. By integrating 3-hydroxybenzo [g]flavone (Fla), MnO_2_, and neutrophil membrane (Neu), Liu et al. [395] fabricated Neu-MnO_2_/Fla platform for the CO controllable releasing and specific anti-inflammation. As illustrated in Fig. 10c, the MnO_2_ NPs modified with neutrophil membrane endowed Neu-MnO_2_/Fla platform favorable targeted ability. Herein, hollow mesoporous MnO_2_ NPs not only acted as ideal carrier for their superior drug-loading capacity and brilliant biodegradability, but also could decompose endogenous H_2_O_2_ and facilitated in situ CO release under light owing to the CAT-like ability, thereby achieving synergistic anti-inflammatory. The decrease of local ROS level and pro-inflammatory cytokines (tumor necrosis factor-α, TNF-α and Interleukin-1β, IL-1β) in a lipopolysaccharide (LPS)-induced inflammation model has indicated the effectiveness and controllability of Neu-MnO_2_/Fla platform.

Despite the tremendous attention that paid to nanozyme-related anti-inflammatory therapies, there are still few reports about metal- and metal oxide-based nanozymes with reactive nitrogen species (RNS) scavenging ability. RNS including nitric oxide (^**·**^NO), nitrogen dioxide (^**·**^NO_2_) and peroxynitrite (^**·**^ONOO^−^) etc. are a major culprit in aggravating neuroinflammation induced by traumatic brain injury (TBI) [401]. Miao et al. prepared polyethylene glycol (PEG) coated (PEGylated) ultra-small rhodium nanodots (Rh-PEG NDs) showing excellent multi-enzyme-like activity and high photothermal conversion efficiency [396]. On the one hand, Rh-PEG NDs possessed similar RONS removal capacity as natural CAT, thereby alleviating the inflammation of colon disease. On the other hand, they could be used for photoacoustic imaging and photothermal therapy (Fig. 10d). Mu et al. [52] prepared PtPdMo trimetallic (triM) nanozymes for neuroinflammation treatment through multi-enzyme mimetics reaction-based RONS elimination. In addition, triM nanozymes displayed highly catalytic selectivity in neutral environments, which provided possible application of nanozymes in brain science (Fig. 10e). Zhang et al. doped Cr^3+^ ions into CeO_2_ to prepare Cr/CeO_2_ nanozymes by increasing Ce^3+^ states [402]. The higher Ce^3+^/Ce^4+^  ratio contributed to strengthening  enzyme-like activity of nanozymes with 3–5 times higher than undoped CeO_2_. The  Cr/CeO_2_-based catalytic patch has been demonstrated as a promising choice for non-invasive TBI treatment and neuroinflammation relief owing to the satisfactory RONS (including ^**·**^OH, ONOO^−^ and H_2_O_2_) scavenging ability.

### Application in Cancer Treatment

According to the latest global cancer statistics from the World Health Organization/International Cancer Center team, cancer is expected to become the main cause of death in countries around the world in twenty-first century [403]. Compared with traditional tumor treatment methods (surgery, chemotherapy, radiotherapy, etc.), external minimally invasive or non-invasive strategies containing photodynamic therapy (PDT), chemodynamic therapy (CDT), sonodynamic therapy (SDT), immunotherapy etc. show a favorable development prospect due to their accurate tumor specificity, space/time controllability and biosafety [404, 405]. However, the complex tumor microenvironment (TME) limited the therapeutic effects of many methods. TME not only refers to structure, function and metabolism of tumor tissue, but is also related to the internal environment of tumor cell (nuclear and cytoplasm) possessing the characteristics of hypoxia, acidity, glutathione and overexpression of H_2_O_2_ [406, 407]. The intrinsic catalytic activity enables nanozymes to regulate TME via changing RONS content or eliminating hypoxia [43, 408–410]. The biological safety, photothermal performance and some other physicochemical properties of nanozymes also indicated their potential in cancer therapy [411]. Given these reasons, nanozymes have been regarded as the prospective standalone agents or synergist for the progress of tumor treatment [43].

#### Photodynamic Therapy

**Fig. 11:**
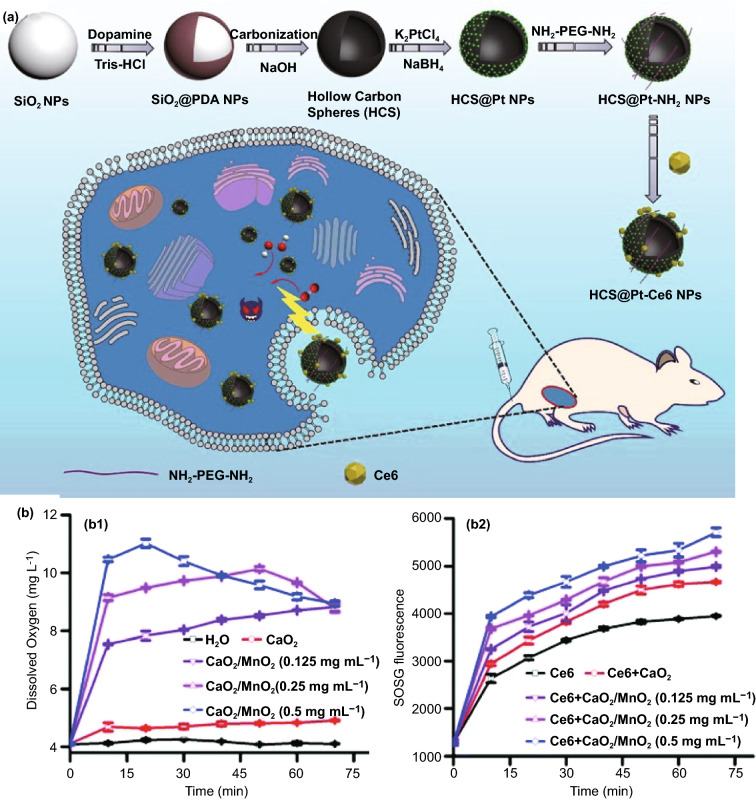
**a** Synthesis progress of HCS@Pt-Ce6 NPs with multi-enzyme-like activity for PDT enhancement. The content of produced b**1** O_2_ and **b2** ^1^O_2_ (via Ce6) with CaO_2_ NPs at 0.5 mg mL^−1^ and MnO_2_ NPs at different concentrations. Adapted from **a** Ref. [415], **b** Ref. [416] with permission

PDT relied on ROS generated by photosensitizers (PSs) under light irradiation to induce cancer cell apoptosis [412]. Nevertheless, most PSs still face disadvantages of low selectivity, poor water solubility and high self-destruction [413]. In order to reinforce the stability of loading PSs, various nanozymes such as MnO_2_ [414], Pt[51] and so on were utilized. In the research of Xu et al. [415], Pt/C nanozymes not only served as chlorin e6 (Ce6) nanocarriers, but also promoted the conversion of H_2_O_2_ and O_2_ into ROS with anti-tumor property (Fig. 11a). They compared the nanozymes with various structures and found that HCS@Pt NPs (Pt NPs decorated with hollow carbon spheres) showed favorable POD- and OXD-like activity, thereby further firming the therapeutic efficacy of PDT for cancer.

In addition to PSs transportation, studies have also confirmed that tumor hypoxia would weaken PDT efficiency [417]. Hence, nanozymes (e.g., Pt [418], Mn_3_O_4_[419]) as CAT mimics were employed to consume intratumoral H_2_O_2_ and generate oxygen in parallel with photosensitizer carriage. However, tumor hypoxia was difficult to be continuously suppressed due to the respiration of intratumoral mitochondria [420]. Yang et al. integrated IR780 PSs into mesoporous silica NPs (MSNs) and then covered with Mn_3_O_4_ NPs to produce Mn_3_O_4_@MSNs@IR780 nanocomposites [419]. Mn_3_O_4_ nanozymes that accumulated in the tumor sites could decompose H_2_O_2_ and trigger switch to release IR780, which specifically targeted to mitochondria and produced ROS to inhibit cancer cells respiration after destroying mitochondria. In vitro experiments proved that oxygen supplementation and mitochondrial destruction were vital to PDT enhancement. Hu et al. [416] employed exogenous oxygen-generating materials (CaO_2_ NPs) to alleviate tumor hypoxia. In this report, MnO_2_ nanozymes with CAT-mimicking activity not only catalyzed CaO_2_ NPs to generate O_2_, but also allowed image-guided PDT as a promising MR T1 nanoprobe (Fig. 11b).

#### Chemodynamic Therapy

Chemodynamic therapy generates ^**·**^OH by catalyzing intratumoral H_2_O_2_ via Fenton or Fenton-like reactions, thereby killing tumor cells [421]. Nanozymes with POD-like activity (e.g., Fe_3_O_4_ NPs [422], AFeNPs [423]) have been recognized as Fenton reaction catalysts for CDT in acidic environments. Since existing reports revealed the pH-dependence of CDT, the pH-independent nanozymes (e.g., Fe/Al-GNE [424], Au_2_Pt [142]) were designed to provide efficient Fenton reactions in neutral TME. What’s worse, high concentration of GSH and low H_2_O_2_ in TME have also been demonstrated to restricted CDT effect [425]. Therefore, conquering the above-mentioned TME is a challenge to optimize CDT reaction efficiency.

**Fig. 12:**
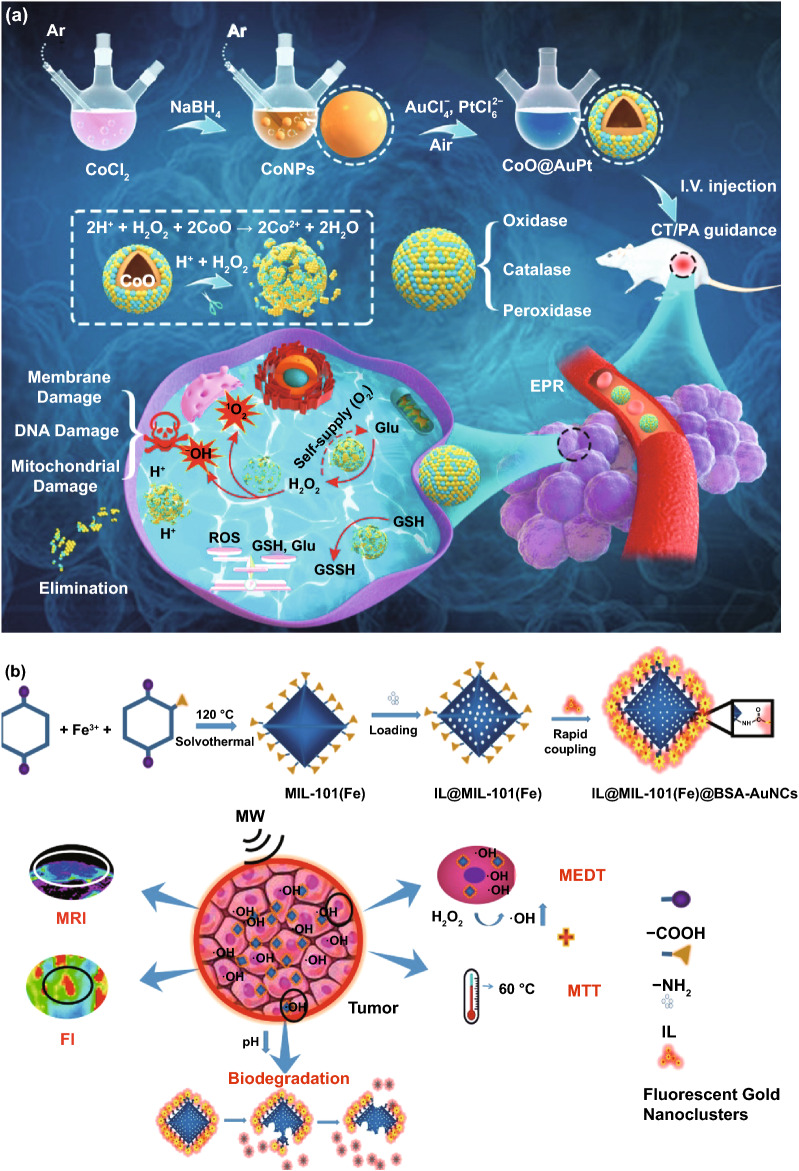
**a** Preparation and the catalytic mechanism for CDT enhancement of CoO@AuPt NPs via Fenton reactions and regulating the response environment. **b** Preparation, the degradation process and the therapy principle of IL@MIL-101(Fe)@BSA-AuNCs NPs for MEDT. *GSSH* Glutathione disulfide, *EPR* enhanced permeation and retention, *MW* microwave, *MRI* magnetic resonance imaging, *MTT* microwave thermal therapy, *FI* fluorescence imaging. Adapted from **a** Ref. [426], **b** Ref. [131] with permission

Fu et al. synthesized CoO@AuPt nanocatalyst with high biocompatibility and stability under physiological environment, which regulated responsive CDT by lowering pH, increasing H_2_O_2_ level and consuming GSH content [426]. In the work, CoO template could degrade and generate Co^2+^ in acidic and high-level H_2_O_2_ environment, which was further acted as a useful Fenton-like reagent. The released Au/Pt nanozymes as multi-enzyme (GPx, CAT, POD, and GOx) mimics were responsible for decreasing GSH concentration and catalyzing H_2_O_2_ into O_2_ and ^**·**^OH (Fig. 12a). Moreover, the nanosatellites consumed intratumoral glucose to generate numerous H_2_O_2_ and induced starvation therapy, thereby enhancing the effect of CDT.

Another challenge to achieve augmented CDT is to increase the generation rate of ^**·**^OH. Ma et al. [131] introduced microwave (MW) as an external stimulus to regulate CDT and realize controllable tumor therapy, named as microwave enhancing dynamic-therapy (MEDT). By coupling gold nanoclusters (BSA-Au NCs) with Fe-metal organic frameworks (MIL-101(Fe)), IL@MIL-101@BSA-AuNCs NPs were prepared after loading methylimidazolium hexafluorophosphate (IL) on MIL-101(Fe) NPs. Under microwave irradiation, MIL-101(Fe) enzymes owned MEDT by catalyzing H_2_O_2_ to produce toxic ^**·**^OH in tumor. The dynamic distribution of MIL-101 (Fe) NPs in vivo and tumor site could be real-time monitored by magnetic resonance imaging (MRI) and fluorescence imaging (FI) (Fig. 12b).

#### Sonodynamic Therapy

**Fig. 13:**
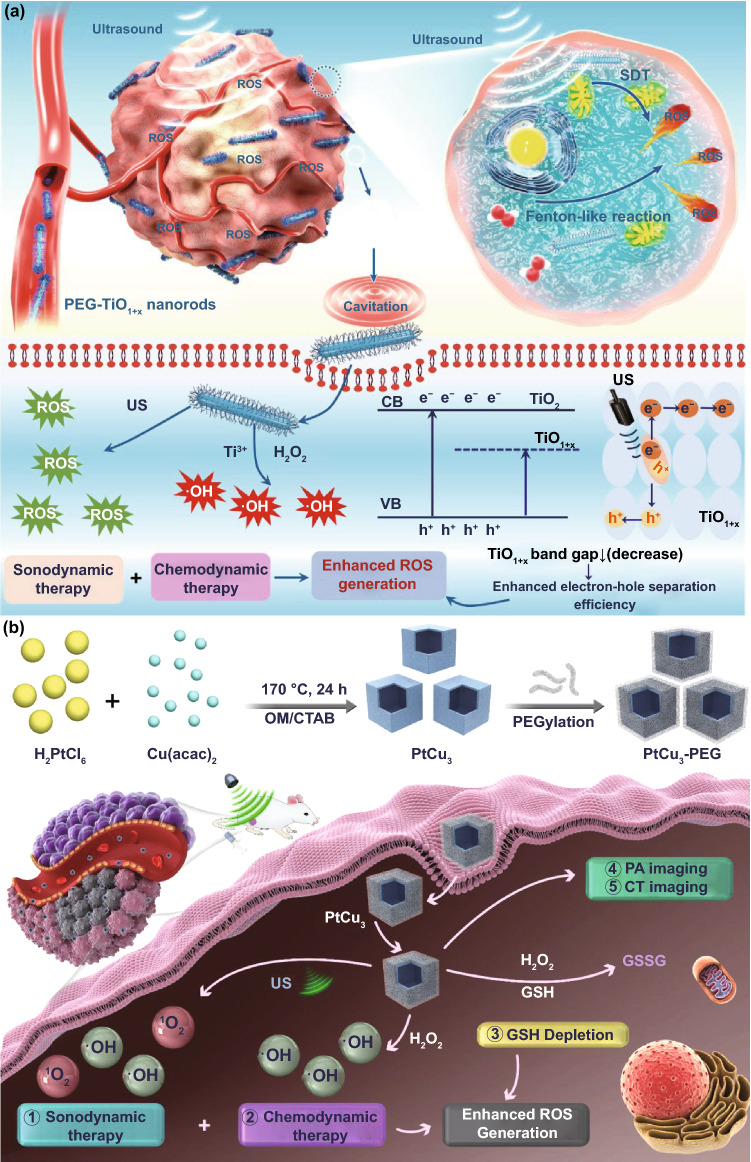
**a** Schematic illustration of the working mechanism of TiO_1 + *x*_ NRs with HRP-like activity for SDT/CDT-combined tumor therapy. **b** Preparation procedure and working mechanism of PtCu_3_-PEG nanocages with HRP- and GPx-type property for PA/CT dual-modal imaging-guided CDT-enhanced SDT. Adapted from **a** Ref. [432], **b** Ref. [433] with permission

PDT is commonly suitable for relatively small superficial tumors due to the limited depth of light penetration through tissues [427]. In contrast, ultrasound (US) owns a higher tissue penetration depth than light waves. Thus, US-triggered sonodynamic therapy is promising to treat deep or large tumors by activating sonosensitizers to generate ROS [428, 429]. Resemble to PSs in PDT, the performance of sonosensitizers plays a fundamental role in SDT [430]. The past 5 years witnessed the development of novel marvelous sonosensitizers [429, 431]. The stability and catalytic activity allowed some metal- and metal oxide-based nanozymes to function as sonosensitizers and Fenton reagents simultaneously to achieve CDT-enhanced SDT. For instance, Wang et al. designed polyethylene glycol (PEG)-modified nanozymes with ultrafine rod-like structure, named PEG-TiO_1+*x*_ NRs for tumor ablation [432]. Compared with traditional inorganic sonosensitizers, the sensitivity of PEG-TiO_1+*x*_ NRs was more prominent due to hypoxic structure. Furthermore, PEG-TiO_1+*x*_ NRs with HRP-type activity showed Fenton-like catalytic property. As SDT reagent possessing CDT function, the intravenously injected PEG-TiO_1+*x*_ NRs were significantly more effective in inhibiting tumors than traditional TiO_2_ NPs under US irradiation (Fig. 13a). Zhong et al. prepared uniform PtCu_3_ nanocages as sensitizers, HRP mimics and GPx mimics by one-step solvothermal method after pegylation [433]. Their research confirmed that PtCu_3_ for cancer therapy improved sound toxicity and inhibited tumor growth by generating ROS by decomposing H_2_O_2_ into ^**·**^OH and depleting GSH under US, in which PtCu_3_ could obviously optimize the reaction environment of CDT. Meanwhile, owing to high light absorption and strong X-ray attenuation in near-infrared region, PtCu_3_ could be employed for photoacoustic (PA)/computed tomography (CT) imaging-guided CDT-enhanced SDT (Fig. 13b).

#### Photothermal Therapy

**Fig. 14:**
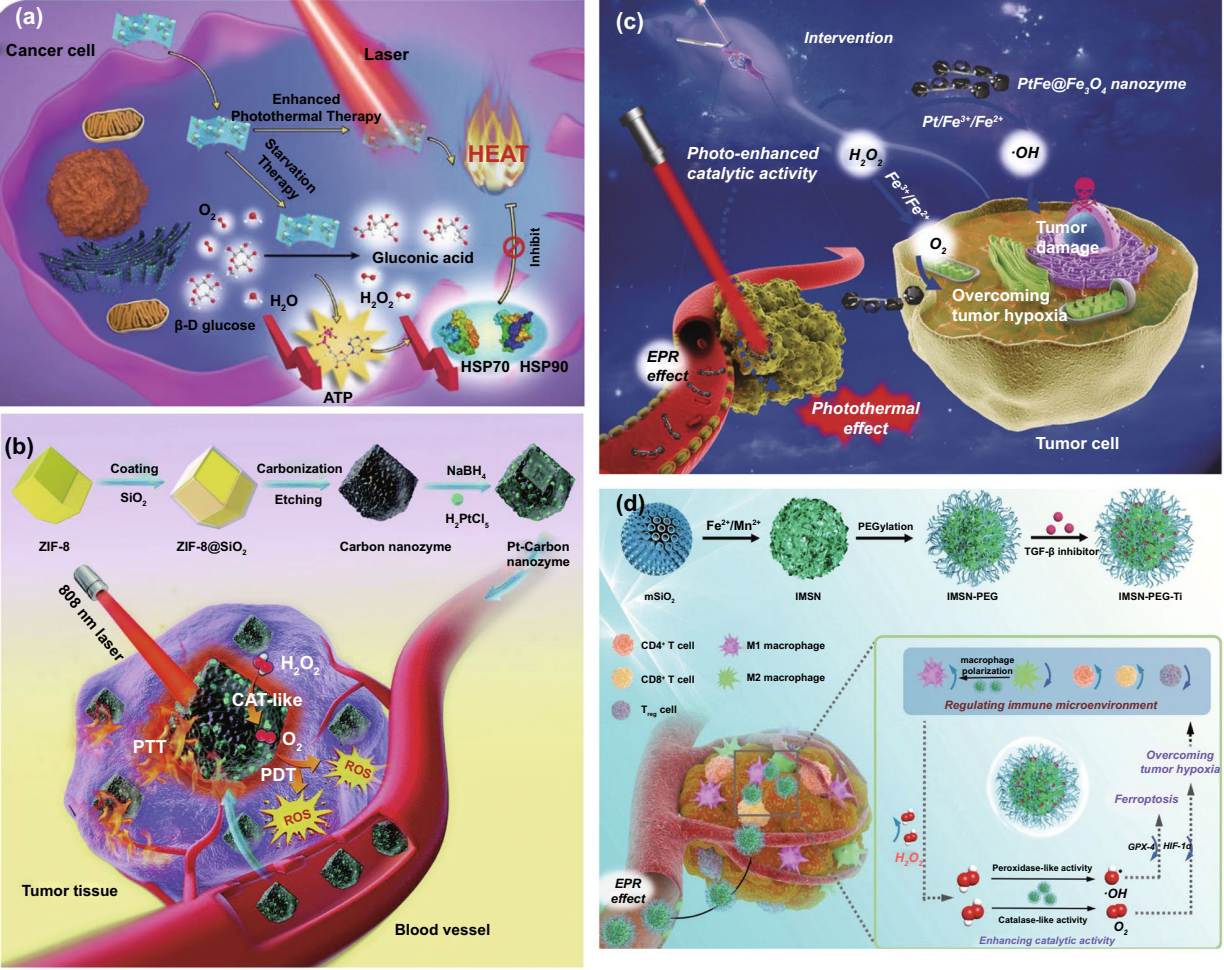
**a** Working principle of M-NSs as GOx mimics and PTAs with effective PA imaging performance for the synergistic starvation-enhanced PTT guided by PA imaging. **b** Synthesis procedure and the working mechanism of Pt-carbon-integrated nanozymes for synergistic PDT/PTT cancer therapy. **c** Working mechanism of PtFe@Fe_3_O_4_ with POD-, CAT-like activity and excellent photothermal effect under acidic TME environment for tumor catalytic therapy combined with PTT. **d** Illustration of the cancer immunotherapy using the IMSN‐PEG‐TI nanoplatform. Adapted from **a** Ref. [435], **b** Ref. [443], **c** Ref. [411] with permission

Materials with high photothermal conversion efficiency are exploited in photothermal therapy (PTT), which could convert light energy into heat energy for the death of cancer cells under external light irradiating [434]. Numerous metal-based and metal oxide-based nanozymes (e.g., MnO_2_ [435], Ru-Te [436], Ru@CeO_2_ [437]) have been reported as photothermal agents (PTAs). In these studies, nanozymes ameliorated PTT efficacy due to their enzyme-like abilities and other superior properties at the same time. Wang et al. [435] synthesized 2D MnO_2_ nanosheets (M-NSs) with controllable protein orientation through a wet chemical method, and then functionalized M-NSs via a sonochemical proposal. As is shown in Fig. 14a, the M-NS served as GOx mimics with highly dispersion and stability, which finally realized starvation therapy by consuming glucose of tumor cells. The nanozymes also presented remarkable photothermal conversion efficiency and PA imaging performance under near-infrared (NIR) irradiation, thereby achieving PA imaging-guided synergistic cancer treatment of starvation therapy and PTT.

However, the effect of PTT is stinted by light penetration depth and thermal damage to healthy tissue induced by overexposure [438]. Therefore, a series of studies tried to combine PTT with other treatment methods to achieve synergistic therapy [439]. For example, Au_2_Pt nanozymes as POD and CAT mimics with potent photothermal performance were reported for PDT/CDT/PTT synergistic cancer therapeutics [142]. AgPd NPs with POD-like activity could improve photothermal conversion efficiency, and have been proved to be acted as carriers for chemotherapeutic drugs transmission during a weakly acidic environment (pH 5.5), thus achieving ROS/PTT/chemotherapy guided by NIR laser [440]. Pt-CuS Janus nanozymes were adopted in synergistically enhanced SDT and PTT [441]. In this system, Pt-CuS Janus hollow structure was used as sonosensitizers carrier, showing photothermal conversion capacity under laser irradiation, and could decompose endogenous H_2_O_2_ expeditiously. The Pt NPs [442] with CAT-mimicking capacity and Ru–Te hollow nanorods [436] with OXD, POD-, CAT- and SOD-type activity both acted as carriers and relieved TME hypoxia to enhance cancer PDT/PTT effect. Different from most nanozyme-based synergistic therapy, Yang et al. [443] covered Pt-carbon integrated nanozymes as PSs via one-step reduction. Under NIR light lasering, the nanozymes provided brilliant photosensitivity and photothermal effect. And the PDT reinforcement was relied on the CAT-like catalysis activity. In vivo experiments revealed that Pt-carbon nanozymes inhibited mice colon cancer reaching an 90% efficiency (Fig. 14b). Li et al. [411] prepared the H_2_O_2_-responsive PtFe@Fe_3_O_4_, which possessed POD-like activity, CAT-type property and exceptional photothermal performance under acidic TME environment. Experimental results indicated that tumor catalytic therapy based on PtFe@Fe_3_O_4_ nanozymes obtained a 99.8% anti-tumor rate for deep pancreatic cancer when cooperating with photothermal therapy What is more, the electron transfer process between PtFe nanorods, Fe_3_O_4_ NPs and H_2_O_2_ molecules was also firstly described in their study (Fig. 14c).

#### Immunotherapy

Cancer immunotherapies, regarded as promising strategies for tumor therapy, utilize the immune system of patients to treat cancer [444], and might include cytokine therapy, tumor vaccines, immune checkpoint blockade (ICB) therapy, adoptive cell therapy and so on [445]. Studies have demonstrated that the modulation of TME is conducive to tumor immunotherapy [43]. Yang et al. [446] designed a polyethylene glycol (PEG)-modified hollow manganese dioxide (H-MnO_2_) nanoshells to load photodynamic agent Ce6 and chemotherapy drug doxorubicin (DOX), forming H-MnO_2_-PEG/C&D complex for cancer combination immunotherapy. The H-MnO_2_ could alleviate tumor hypoxia via catalytically decomposing hydrogen peroxide to generate O_2_. A series of immunological responses were discovered with synergistic treatment of H-MnO_2_-PEG/C&D and Chemo-PDT, resulting remarkable decreasing in the secretion of IL-10 (predominant cytokine secreted by M2 macrophages) and the increment in the secretion of IL-12 (predominant cytokine secreted by M1 macrophages). Moreover, the introduction of anti-PD-L1 checkpoint blockade showed further enhanced therapeutic efficacy for tumor with by improving TNF-α.

Moreover, it has been reported that tumor-associated macrophages (TAMs) are critical to tumor growth and metastasis, thereby playing an important role in the cancer immunotherapy [447]. Regulating TME could facilitate macrophage polarization from M2 to M1 since the tumor hypoxia is associated with macrophage recruitment and polarization [448]. Xu et al. [449] loaded TGF‐β inhibitor (TI) to the PEGylated iron manganese silicate nanoparticles to prepare IMSN-PEG-TI nanoplatform for tumor immunotherapy (Fig. 14d). In this system, IMSN nanozymes with POD- and CAT-like property could decompose H_2_O_2_ into ^**·**^OH and O_2_ to kill tumor cells and overcome tumor hypoxia in respective. The interaction of IMSN and TI effectively regulated the tumor immune microenvironment, leading to elevated ratio of M1 to M2 macrophages, CD4^+^ T to T_reg_ cells, and CD8^+^ T to T_reg_ cells. Furthermore, the enhanced macrophages polarization would in turn induce the reproduction of H_2_O_2_, thus promoting enzymatic properties of IMSN nanozymes.

## Conclusion

The prosperity of nanotechnology and biology created a series of novel artificial enzymes. As promising natural enzymes mimics, nanozymes have demonstrated remarkable performance in clinical medicine, biopharmaceuticals, environmental monitoring and many other fields. In this review, we meticulously elaborated the intrinsic activity and catalytic mechanism of the classical metal- and metal oxide-based nanozymes, including monometal-, metal alloy-, metal oxide-, metallic core/shell nanostructure-based and hybrid nanomaterials. The recent research progress of metal- and metal oxide-based nanozymes in analysis, antibacterial, relieving inflammation, and cancer therapy was also involved. Although nanozymes have been revealed to overcome many limitations of natural enzymes such as low stability, complicated preparation and expensive storage, there are still severe challenges for future researches. (1) Compared with most natural enzymes, metal- and metal oxide-based nanozymes seem to lack the substrate specificity. Even though researchers have discovered amounts of inner and external factors that influencing enzymatic properties, the precise control of catalytic performance, especially for the nanozymes with multi-enzyme-like activities, still has a long way to go. (2) The exploration of the internal catalytic mechanism is fundamental for understanding and mastering the catalytic reaction of nanozymes. In contrast to the synthesis and employment of novel nanomaterials, studies that involved the deep comprehension of working mechanism are relatively rare. What’s worse, the advanced strategies dedicated to mechanism clarification are also limited. (3) The POD mimics have become an issue of extensive concern in most nanozyme-related applications, especially in the field of analysis and detection. While other component of oxidoreductase family have also been proved to possess unsubstituted function in many circumstances. Therefore, the spread utilization of SOD, CAT, OXD mimics are yet to be developed. (4) Most previous biosensors based on nanozymes could only detect one or two substances. The schemes for simultaneous discrimination and quantification of multiple (≥ 3) substances with high sensitivity are required to be further investigated and simplified. (5) Considering the cost control in large-scale preparation, seeking alternatives for noble metal nanozymes has gradually received increasing attention. Besides, the reduction of their content in nanoalloys and nanocomposites while guaranteeing the performance is also worth more efforts. (6) The long-term in vivo toxicity of nanozymes still remains a challenge for their clinical employment. Although a large amount of studies have involved the discussion about the biocompatibility, the systematic mechanisms of toxicity and corresponding solutions are in urgent need.

## Abbreviations

AA: Ascorbic acid
ABTS: 2,2'-Azino-bis(3-ethylbenzothiazoline-6-sulfonic acid)
3-AT: 3-Amino-1,2,4-Triazole
ATP: Adenosine triphosphate
BSA: Bovine serum albumin
CAT: Catalase
CDT: Chemodynamic therapy
Ce6: Chlorine e6
CEA: Carcinoembryonic antigen
CO: Carbon monoxide
CT: Computed tomography
CTP: Cytidine triphosphate
l‑Cys: l‑Cysteine
EPR: Enhanced permeation and retention
ESR: Electron spin resonance
ELISA: Enzyme‐linked immunosorbent assay
GA: Gallic acid
GOx: Glucose oxidase
GPx: Glutathione peroxidase
GSH: Glutathione
GTP: Guanosine triphosphate
H_2_O_2_: Hydrogen peroxide
His: Histidine
HO_2_^·^: Hydroperoxyl radicals
HRP: Horseradish peroxidase
IBD: Inflammatory bowel disease
LFIA: Lateral flow immunoassay
MEDT: Microwave enhancing dynamic-therapy
MNPs: Magnetic nanoparticles
MRI: Magnetic resonance imaging
NCs: Nanoclusters
Neu: Neutrophils
NPs: Nanoparticles
NRs: Nanorods
NSs: Nanosheets
NTP: Nucleoside triphosphate
NWs: Nanowires
O_2_^·^^−^: Oxygen superoxide anion
O_2_^1^: Singlet oxygen
^·^OH: Hydroxyl radical
OOH^−^: Perhydroxyl anion
OPD: *o*-Phenylenediamine
OXD: Oxidase
PA: Photoacoustic
PDT: Photodynamic therapy
PEGylated: Polyethylene glycol
POD: Peroxidase
pNPP: *p*-Nitrophenyl phosphate
PSs: Photosensitizers
PTA: Photothermal agent
PTT: Photothermal therapy
PVP: Polyvinylpyrrolidone
RNS: Reactive nitrogen species
ROS: Reactive oxygen species
RONS: Reactive oxygen or/and nitrogen species
SDT: Sonodynamic therapy
SERS: Raman scattering
SOD: Superoxide dismutase
SPR: Surface plasmon resonance
SuOx: Sulfite oxidase
TA: Tannic acid
TAM: Tumor-associated macrophage
TBI: Traumatic brain injury
TMB: 3,3′,5,5′-Tetramethylbenzidine
TME: Tumor microenvironment
TNF-α: Tumor necrosis factor-α
US: Ultrasound
UTP: Uridine triphosphate
XPS: X-ray photoelectron spectroscopy
